# Supplementary material for: Heat is associated with short-term increases in household food insecurity in 150 countries and this is mediated by income
Source: Nat Hum Behav. 2023 Aug 21;7(10):1777–86. doi: 10.1038/s41562-023-01684-9 (PMC10593604; doi:10.1038/s41562-023-01684-9)
Supplement: Supplementary file 1 — Includes descriptive statistics and robustness checks on the main analyses conducted. [file 41562_2023_1684_MOESM1_ESM.pdf]

nature human behaviour

Article

https://doi.org/10.1038/s41562-023-01684-9

Heat is associated with short-term increases in household food insecurity in 150 countries and this is mediated by income

In the format provided by the authors and unedited

Contents

1 Descriptive statistics **3**

1.1 List of variables . . . 3

1.2 Country profiles with summary statistics . . . 10

1.3 Distribution of food insecurity and heat variable . . . 83

1.4 Distribution of UTCI across day, area, and year . . . 85

2 Main results **86**

2.1 Mediation results . . . 86

2.2 Moderation results . . . 89

3 Experiment **90**

3.1 Covariate balance . . . 90

3.2 Selection bias . . . 92

3.3 Geographic match . . . 94

4 Model specifications **97**

4.1 Non-linearity . . . 97

4.2 Continuous heat measures . . . 99

4.3 Absolute and relative heat measures . . . 101

4.4 Food insecurity measures . . . 107

5 Temporal effects **109**

5.1 Long term effects . . . 109

5.2 Short term lags 113

6 Sensitivity **116**

6.1 Outliers 116

6.2 Covariates 118

1 Descriptive statistics

1.1 List of variables

Data was compiled from three main sources: The ERA5-data sets from the Climate Data Store, the Gallup World Poll for socio-demographic information and food insecurity, and the World Development Indicators by the World Bank for country-level variables.

Table 1: List of variables with sources, scales or units, and definitions.

Variable	Source	Scale or unit	Definition
Food insecurity (FIES)	Gallup World Poll	0-8	During the last 12 months, was there a time when, because of lack of money or other resources:<br>(1) You were worried you would not have enough food to eat?<br>(2) You were unable to eat healthy and nutritious food?<br>(3) You ate only a few kinds of foods?<br>(4) You had to skip a meal?<br>(5) You ate less than you thought you should?<br>(6) Your household ran out of food?<br>(7) You were hungry but did not eat?<br>(8) You went without eating for a whole day?
Mild-severe food insecurity	Gallup World Poll	0,1	At least 1 'yes' response to the FIES

Variable	Source	Scale or unit	Definition
Moderate-severe food insecurity	Gallup World Poll	0,1	At least 4 'yes' responses to the FIES
Severe food insecurity	Gallup World Poll	0,1	At least 7 'yes' responses to the FIES
Hot week	ERA-5 HEAT CDS Climate Data Store	0,1	Whether the previous seven days on a given survey date included at least 3 days that are in the hottest 10 percent of the year for that sub-region
Area	Gallup World Poll	Urban<br>Rural	Area in which a respondent lives
Children	Gallup World Poll	Yes<br>No	Whether any children under 15 are living in the household
Age	Gallup World Poll	Years	Age of respondent
Partner	Gallup World Poll	Yes<br>No	Whether respondent has a partner (including married and unmarried couples)
Gender	Gallup World Poll	Female<br>Male	Gender respondent reported

Variable	Source	Scale or unit	Definition
Employment	Gallup World Poll	0,1	Whether a respondent was unemployed or underemployed in the last seven days
Annual income	Gallup World Poll	Internationl USD, (PPP) ln	Annual household income before taxes calculated from the monthly household income reported by the respondent. Includes wages, salaries, remittances from family members living elsewhere, farming, and all other sources
Health problems	Gallup World Poll	0,1	Whether a respondent has any health problems that prevent them from doing any of the things people their age normally can do
Feelings about income	Gallup World Poll	0,1	Whether a respondent finds it difficult or very difficult on their present income compared to living comfortably or getting by on their income
Local job market	Gallup World Poll	0,1	Whether a respondent described the local job market as a 'Good time' (= 0) or a 'Bad time' (= 1)
Average year-round UTCI in sub-region	ERA-5 HEAT CDS Climate Data Store	ºC	Universal Thermal Comfort Index. Composite measure of temperature, humidity, wind, and radiation. Calculated as the average of daily mean UTCI in a subregion in a year

Variable	Source	Scale or unit	Definition
Precipitation	NOAA PSL CPC	mm, ln	Daily total of precipitation over a 0.5 x 0.5 longitude and latitude grid
GNI per capita	World Development Indicators	current USD	Gross national income per capita, Atlas method
Year	Gallup World Poll	2014-2017	Year of survey date
Hot days in a year	ERA-5 HEAT CDS Climate Data Store	7-65	Number of days in the hottest 10% of the year in that sub-region
Agricultural employment	World Development Indicators (ILO)	% of total employment	Agricultural employment in a country
Vulnerable employment	World Development Indicators (ILO)	% of total employment	Vulnerable employment includes contributing family workers and own-account workers
Wage and salaried labour	World Development Indicators (ILO)	% of total employment	Wage and salaried workers hold explicit or implicit employment contracts that give them a basic remuneration that is not directly dependent upon the revenue of the unit for which they work.

Variable	Source	Scale or unit	Definition
Formality	World Development Indicators (ILO)	% of firms	Firms formally registered when operations started

Table 2: Descriptive Statistics: Continuous variables

**Variable**	**n**	**Min**	$\mathbf{q_1}$	$\tilde{x}$	$\bar{x}$	$\mathbf{q_3}$	**Max**	**s**	**#NA**
Age	587941	15.0	27.0	40.0	42.2	55.0	101.0	17.8	1741
Precipitation in week (ln)	480991	-42.8	-1.4	0.3	-2.0	1.2	4.1	8.2	108691
Average year-round UTCI	589682	-11.2	8.7	16.5	16.2	25.3	34.2	9.5	0
GNI per capita (ln)	579647	5.6	7.6	8.8	8.8	10.0	11.6	1.4	10035
Vulnerable employment	575806	0.1	10.8	27.6	34.7	52.3	94.3	26.8	13876
Agricultural employment	575806	0.1	4.7	17.3	22.8	33.9	86.2	20.9	13876
Formality	155889	57.7	83.5	87.2	85.8	91.4	97.2	8.8	433793

Table 3: Descriptive statistics for categorical variables

**Variable**	**Levels**	**n**	%
Hot week	No	501276	85.0
	Yes	88392	15.0
	all	589668	100.0
Severe food insecurity	No	471779	86.8
	Yes	72083	13.2
	all	543862	100.0
Moderate-to-severe food insecurity	No	403742	74.2
	Yes	140120	25.8
	all	543862	100.0
Mild-to-severe food security	No	300672	55.3
	Yes	243190	44.7
	all	543862	100.0
Area	Rural	339277	57.9
	Urban	246476	42.1
	all	585753	100.0
Children under 15 in household	No	289951	49.4
	Yes	296526	50.6
	all	586477	100.0
Having a partner	No	243428	41.6
	Yes	341852	58.4
	all	585280	100.0
Gender: Male	Female	313466	53.2
	Male	276216	46.8
	all	589682	100.0
Year	2014	170542	28.9
	2015	137745	23.4
	2016	140502	23.8
	2017	140893	23.9
	all	589682	100.0

1.2 Country profiles with summary statistics

Table 4 supports the interpretation and applicability of results for researchers and policy makers. The table provides country profiles with the summary statistics for a hot week, daily Universal Thermal Climate Index, as well as agricultural employment as a share of total employment, vulnerable employment as a share of total employment, formality as the share of firms formally registered, the share of waged or salaried labour as a share of total employment, and the gross national income per capita in a logarithm. The statistics also include the average absolute UTCI value that underlies the 90th percentile of UTCI in the sub-regions within the country. This table can be used to gauge whether a country would be expected to experience impacts of a hot week on food insecurity.

Table 4: Country profiles

Variable	NotNA	Mean	Median	Sd	Min	Pctile[25]	Pctile[75]	Max
Country: Afghanistan								
Hot week	3815	0.056	0	0.23	0	0	0	1
Threshold at 90th percentile	3815	25	25	5.5	12	21	29	35
Daily UTCI	3815	16	17	8.9	-16	10	23	36
Agricultural employment	3815	46	46	1.7	44	44	48	48
Vulnerable employment	3815	82	81	1.3	80	80	83	83
Formality	3815	92	92	0	92	92	92	92
Wage labour	3815	16	16	0.91	15	15	18	18
GNI per capita (ln)	3815	6.4	6.3	0.068	6.3	6.3	6.4	6.4
Country: Albania								

Hot week	3998	0.36	0	0.48	0	0	1	1
Threshold at 90th percentile	3998	26	27	2.5	19	25	28	30
Daily UTCI	3998	24	24	3.8	8.3	21	26	31
Agricultural employment	3938	39	40	5.9	5.7	38	41	42
Vulnerable employment	3938	54	56	7.5	11	54	56	56
Formality	0				Inf			-Inf
Wage labour	3938	43	42	6.7	41	41	43	81
GNI per capita (ln)	3998	8.4	8.4	0.18	8.3	8.4	8.4	10
				Country: Algeria				
Hot week	2959	0	0	0	0	0	0	0
Threshold at 90th percentile	2959	27	27	1.7	24	26	28	35
Daily UTCI	2959	18	19	5.1	7.5	15	23	28
Agricultural employment	2959	10	10	0.14	10	10	10	10
Vulnerable employment	2959	27	27	0.066	27	27	27	27
Formality	0				Inf			-Inf
Wage labour	2959	69	68	0.22	68	68	69	69
GNI per capita (ln)	2959	8.4	8.4	0.14	8.3	8.3	8.6	8.6
				Country: Angola				
Hot week	978	0	0	0	0	0	0	0

Threshold at 90th percentile	978	26	25	3.1	22	24	30	32
Daily UTCI	978	20	21	2.5	13	17	21	25
Agricultural employment	978	51	51	0	51	51	51	51
Vulnerable employment	978	70	70	0	70	70	70	70
Formality	0				Inf			-Inf
Wage labour	978	26	26	0	26	26	26	26
GNI per capita (ln)	978	8.5	8.5	0	8.5	8.5	8.5	8.5
				Country: Argentina				
Hot week	3975	0	0	0	0	0	0	0
Threshold at 90th percentile	3975	26	25	2.6	13	25	27	31
Daily UTCI	3975	7.3	7.3	6	-11	3.4	11	28
Agricultural employment	3975	0.25	0.13	0.19	0.06	0.06	0.55	0.55
Vulnerable employment	3975	21	21	0.53	20	20	22	22
Formality	3975	92	92	0	92	92	92	92
Wage labour	3975	76	75	0.66	75	75	76	76
GNI per capita (ln)	3975	9.4	9.4	0.028	9.4	9.4	9.5	9.5
				Country: Armenia				
Hot week	3928	0.36	0	0.48	0	0	1	1
Threshold at 90th percentile	3928	22	22	2.7	16	19	23	27

Daily UTCI	3928	20	20	3.7	9.5	17	22	31
Agricultural employment	3928	32	34	7.9	5.9	31	35	37
Vulnerable employment	3928	39	41	11	5.3	39	43	57
Formality	0				Inf			-Inf
Wage labour	3928	60	58	12	32	56	60	93
GNI per capita (ln)	3928	8.4	8.3	0.31	8.2	8.3	8.3	9.6
Country: Australia								
Hot week	4977	0.0016	0	0.04	0	0	0	1
Threshold at 90th percentile	4977	24	23	3.1	15	22	26	33
Daily UTCI	4977	14	14	6.3	-7.7	9.7	18	33
Agricultural employment	4977	2.7	2.6	0.089	2.6	2.6	2.8	2.8
Vulnerable employment	4977	11	11	0.048	11	11	11	11
Formality	0				Inf			-Inf
Wage labour	4977	83	83	0.088	83	83	83	83
GNI per capita (ln)	4977	11	11	0.097	11	11	11	11
Country: Austria								
Hot week	4942	0.057	0	0.23	0	0	0	1
Threshold at 90th percentile	4942	20	20	2.6	15	18	21	24
Daily UTCI	4942	7.9	9.3	8.7	-17	0.73	15	27

Agricultural employment	4942	4.5	4.5	0.34	3.9	4.3	4.8	4.8
Vulnerable employment	4942	8.3	8.5	0.33	7.7	8.2	8.6	8.6
Formality	0				Inf			-Inf
Wage labour	4942	87	87	0.35	87	87	87	88
GNI per capita (ln)	4942	11	11	0.045	11	11	11	11
			Country: Bahrain					
Hot week	3009	0	0	0	0	0	0	0
Threshold at 90th percentile	3009	37	36	0.94	36	36	38	38
Daily UTCI	3009	26	31	9.8	1.1	16	33	35
Agricultural employment	3009	1.1	1.1	0	1.1	1.1	1.1	1.1
Vulnerable employment	3009	1	1.1	0.02	1	1	1.1	1.1
Formality	0				Inf			-Inf
Wage labour	3009	97	97	0.01	97	97	97	97
GNI per capita (ln)	3009	10	10	0.032	10	10	10	10
			Country: Bangladesh					
Hot week	3965	0.11	0	0.31	0	0	0	1
Threshold at 90th percentile	3965	33	33	0.45	32	33	33	34
Daily UTCI	3965	30	30	2.3	23	29	32	35
Agricultural employment	3965	43	43	1.5	41	43	44	46

Vulnerable employment	3965	61	59	6.3	56	58	60	78
Formality	509	87	87	0	87	87	87	87
Wage labour	3965	37	39	6	20	39	39	40
GNI per capita (ln)	3965	7.2	7.2	0.14	7	7.1	7.3	7.5
Country: Belarus								
Hot week	4144	0.26	0	0.44	0	0	1	1
Threshold at 90th percentile	4144	19	20	1.6	17	18	21	22
Daily UTCI	4144	15	15	5.4	0.58	11	20	27
Agricultural employment	4144	10	9.9	0.41	9.7	9.7	11	11
Vulnerable employment	4144	3.3	3.3	0.085	3.3	3.3	3.5	3.5
Formality	0				Inf			-Inf
Wage labour	4144	96	96	0.16	96	96	96	96
GNI per capita (ln)	4144	8.7	8.6	0.14	8.6	8.6	8.8	8.9
Country: Belgium								
Hot week	5010	0.11	0	0.31	0	0	0	1
Threshold at 90th percentile	5010	19	19	0.71	16	18	19	20
Daily UTCI	5010	11	12	6.3	-13	6.7	15	27
Agricultural employment	5010	1.2	1.2	0.032	1.2	1.2	1.2	1.2
Vulnerable employment	5010	11	11	0.23	10	11	11	11

Formality	0				Inf			-Inf
Wage labour	5010	85	85	0.3	85	85	85	86
GNI per capita (ln)	5010	11	11	0.048	11	11	11	11
			Country: Belize					
Hot week	486	0	0	0	0	0	0	0
Threshold at 90th percentile	486	30	30	0.6	29	30	31	31
Daily UTCI	486	25	25	2.6	20	23	27	29
Agricultural employment	486	18	18	1.6	14	18	18	18
Vulnerable employment	486	24	24	1.5	24	24	24	28
Formality	0				Inf			-Inf
Wage labour	486	69	69	0.51	68	69	69	69
GNI per capita (ln)	486	8.5	8.4	0.32	8.4	8.4	8.4	9.3
			Country: Benin					
Hot week	3852	0.24	0	0.43	0	0	0	1
Threshold at 90th percentile	3852	32	31	0.83	30	31	32	35
Daily UTCI	3852	29	30	2.6	24	28	31	37
Agricultural employment	3852	41	41	1.1	36	40	41	42
Vulnerable employment	3852	88	88	1.5	77	88	88	88
Formality	3852	89	91	7.4	58	91	91	91

Wage labour	3852	11	11	1.7	10	11	11	21
GNI per capita (ln)	3852	7.1	7.1	0.19	6.5	7	7.1	8
Country: Bhutan								
Hot week	2040	0.48	0	0.5	0	0	1	1
Threshold at 90th percentile	2040	20	20	3.6	11	18	23	29
Daily UTCI	2040	20	20	3.8	11	17	23	31
Agricultural employment	2040	57	57	2.8	45	57	58	58
Vulnerable employment	2040	72	72	1.6	71	71	72	78
Formality	2040	95	95	1.9	87	95	95	95
Wage labour	2040	28	28	1.9	20	28	29	29
GNI per capita (ln)	2040	7.8	7.8	0.11	7.4	7.8	7.8	7.8
Country: Bolivia								
Hot week	3981	0.076	0	0.27	0	0	0	1
Threshold at 90th percentile	3981	18	17	7.7	9.4	9.7	27	31
Daily UTCI	3981	13	11	8.1	-8.5	6	19	31
Agricultural employment	3981	30	30	1.2	28	28	31	31
Vulnerable employment	3981	60	57	4.4	55	55	64	65
Formality	3981	80	80	0	80	80	80	80
Wage labour	3981	34	37	3.7	30	31	39	39

GNI per capita (ln)	3981	8	8	0.033	7.9	7.9	8	8
Country: Bosnia and Herzegovina								
Hot week	3737	0.1	0	0.3	0	0	0	1
Threshold at 90th percentile	3737	23	23	1.9	18	22	25	27
Daily UTCI	3737	19	20	3.6	4.5	18	22	27
Agricultural employment	3737	17	17	0.44	17	17	18	18
Vulnerable employment	3737	19	19	0.34	18	18	19	19
Formality	0				Inf			-Inf
Wage labour	3737	76	76	0.57	75	76	77	77
GNI per capita (ln)	3737	8.6	8.5	0.014	8.5	8.5	8.6	8.6
Country: Botswana								
Hot week	3580	0	0	0	0	0	0	0
Threshold at 90th percentile	3580	27	27	1.5	24	25	27	30
Daily UTCI	3580	17	16	5.5	5.8	13	23	29
Agricultural employment	3580	20	21	6.4	4.7	21	22	53
Vulnerable employment	3580	21	23	6.9	8.8	22	23	77
Formality	0				Inf			-Inf
Wage labour	3580	76	75	6	22	75	75	86
GNI per capita (ln)	3580	8.8	8.8	0.15	7.2	8.8	8.9	8.9

Country: Brazil

Hot week	3999	0.031	0	0.17	0	0	0	1
Threshold at 90th percentile	3999	27	27	2.4	23	25	30	32
Daily UTCI	3999	21	22	5.3	2.9	18	25	32
Agricultural employment	3999	10	10	0.34	9.5	10	10	10
Vulnerable employment	3999	27	27	0.65	26	26	27	28
Formality	0				Inf			-Inf
Wage labour	3999	69	69	0.85	68	68	70	70
GNI per capita (ln)	3999	9.2	9.2	0.13	9.1	9.1	9.4	9.4

Country: Bulgaria

Hot week	3980	0.31	0	0.46	0	0	1	1
Threshold at 90th percentile	3980	24	24	1.6	21	23	25	28
Daily UTCI	3980	21	21	4	4.1	19	24	31
Agricultural employment	3980	6.9	6.9	0.11	6.8	6.8	7	7
Vulnerable employment	3980	8.4	8.2	0.2	8.2	8.2	8.5	8.7
Formality	0				Inf			-Inf
Wage labour	3980	88	88	0.28	88	88	88	88
GNI per capita (ln)	3980	8.9	8.9	0.017	8.9	8.9	8.9	8.9

Country: Burkina Faso

Hot week	3496	0.33	0	0.47	0	0	1	1
Threshold at 90th percentile	3496	33	34	0.7	30	33	34	35
Daily UTCI	3496	32	32	2.2	24	31	33	37
Agricultural employment	3496	29	29	0.88	28	28	30	30
Vulnerable employment	3496	86	86	0.56	86	86	87	87
Formality	0				Inf			-Inf
Wage labour	3496	13	13	0.56	12	13	14	14
GNI per capita (ln)	3496	6.6	6.5	0.048	6.5	6.5	6.6	6.6

Country: Burundi

Hot week	846	0.22	0	0.41	0	0	0	1
Threshold at 90th percentile	846	22	22	1.5	20	21	24	25
Daily UTCI	846	21	22	1.8	17	20	23	25
Agricultural employment	846	86	86	0	86	86	86	86
Vulnerable employment	846	84	84	0	84	84	84	84
Formality	846	87	87	0	87	87	87	87
Wage labour	846	15	15	0	15	15	15	15
GNI per capita (ln)	846	5.6	5.6	0	5.6	5.6	5.6	5.6

Country: Cambodia

Hot week	4573	0.13	0	0.33	0	0	0	1
Threshold at 90th percentile	4573	33	33	1.1	30	32	34	36
Daily UTCI	4573	31	32	2	25	30	33	35
Agricultural employment	4573	41	38	3.5	31	38	42	46
Vulnerable employment	4573	52	51	2.6	48	50	52	62
Formality	4573	71	70	4.8	70	70	70	92
Wage labour	4573	47	49	2.8	36	48	50	50
GNI per capita (ln)	4573	7.1	7	0.24	6.9	7	7.1	8.7
			Country: Cameroon					
Hot week	3837	0.32	0	0.46	0	0	1	1
Threshold at 90th percentile	3837	29	28	3.1	24	27	32	34
Daily UTCI	3837	28	27	2.8	21	26	30	34
Agricultural employment	3837	46	46	0.99	45	45	47	48
Vulnerable employment	3837	74	73	0.69	73	73	74	74
Formality	3837	81	81	0	81	81	81	81
Wage labour	3837	23	24	0.68	23	23	24	24
GNI per capita (ln)	3837	7.3	7.3	0.039	7.2	7.2	7.3	7.3
			Country: Canada					
Hot week	4992	0.11	0	0.31	0	0	0	1

Threshold at 90th percentile	4993	20	20	2.4	13	19	21	24
Daily UTCI	4993	8.5	12	10	-23	2	17	27
Agricultural employment	4993	1.6	1.6	0.074	1.5	1.6	1.7	1.7
Vulnerable employment	4993	11	11	0.038	11	11	11	11
Formality	0				Inf			-Inf
Wage labour	4993	85	85	0.045	85	85	85	85
GNI per capita (ln)	4993	11	11	0.083	11	11	11	11
				Country: Central African Republic				
Hot week	1782	0.067	0	0.25	0	0	0	1
Threshold at 90th percentile	1782	31	31	0.68	29	30	31	32
Daily UTCI	1782	29	29	1.5	25	28	30	31
Agricultural employment	1782	71	71	0.26	71	71	71	71
Vulnerable employment	1782	93	93	0.06	93	93	93	93
Formality	0				Inf			-Inf
Wage labour	1782	6.6	6.6	0.05	6.5	6.5	6.6	6.6
GNI per capita (ln)	1782	6.1	6.1	0.035	6	6	6.1	6.1
				Country: Chad				
Hot week	3851	0.16	0	0.37	0	0	0	1
Threshold at 90th percentile	3851	34	34	0.87	32	33	34	35

Daily UTCI	3851	31	31	2	26	29	33	36
Agricultural employment	3851	74	76	6.3	45	75	76	76
Vulnerable employment	3851	90	90	3.8	73	90	91	92
Formality	189	81	81	0	81	81	81	81
Wage labour	3851	9.4	9.4	3.2	7.6	8.2	9.5	24
GNI per capita (ln)	3851	6.7	6.8	0.21	6.5	6.6	6.9	7.3
				Country: Chile				
Hot week	4018	0.017	0	0.13	0	0	0	1
Threshold at 90th percentile	4018	20	22	4	5.4	19	23	23
Daily UTCI	4018	10	9.5	6.7	-13	5.7	15	23
Agricultural employment	4018	8.9	9.4	1.9	0.06	9.2	9.4	9.5
Vulnerable employment	4018	22	22	0.65	20	21	22	23
Formality	190	92	92	0	92	92	92	92
Wage labour	4018	74	74	0.79	73	74	74	76
GNI per capita (ln)	4018	9.5	9.5	0.052	9.4	9.5	9.6	9.6
				Country: China				
Hot week	16988	0.2	0	0.4	0	0	0	1
Threshold at 90th percentile	16988	29	30	3.5	21	28	32	34
Daily UTCI	16988	23	24	7	-7.4	19	29	36

Agricultural employment	16988	28	29	0.95	27	28	30	30
Vulnerable employment	16988	45	45	0.52	45	45	46	46
Formality	0				Inf			-Inf
Wage labour	16988	53	53	0.46	52	52	53	53
GNI per capita (ln)	16988	9	9	0.054	8.9	8.9	9	9.1
			Country: Colombia					
Hot week	3884	0.057	0	0.23	0	0	0	1
Threshold at 90th percentile	3884	24	25	6.7	14	17	31	35
Daily UTCI	3884	23	24	7.2	9.1	15	30	34
Agricultural employment	3884	16	16	0.21	16	16	16	17
Vulnerable employment	3884	47	47	0.16	47	47	47	47
Formality	3884	91	91	0	91	91	91	91
Wage labour	3884	49	49	0.18	48	48	49	49
GNI per capita (ln)	3884	8.8	8.8	0.12	8.7	8.8	9	9
			Country: Congo (Kinshasa)					
Hot week	3961	0.13	0	0.33	0	0	0	1
Threshold at 90th percentile	3961	30	30	1.4	21	29	31	32
Daily UTCI	3961	28	28	2.9	20	26	30	33
Agricultural employment	3961	66	66	0.52	65	66	67	67

Vulnerable employment	3961	78	78	0.26	78	78	79	79
Formality	0				Inf			-Inf
Wage labour	3961	20	20	0.2	19	19	20	20
GNI per capita (ln)	3961	6.1	6.1	0.024	6.1	6.1	6.2	6.2
Country: Congo Brazzaville								
Hot week	3503	0.17	0	0.38	0	0	0	1
Threshold at 90th percentile	3503	30	30	0.71	28	30	31	31
Daily UTCI	3503	27	27	2.5	21	25	29	32
Agricultural employment	3503	34	34	0.3	34	34	34	35
Vulnerable employment	3503	77	77	1	75	76	77	77
Formality	0				Inf			-Inf
Wage labour	3503	23	22	1.1	22	22	23	24
GNI per capita (ln)	3503	7.8	7.7	0.32	7.4	7.4	8	8.3
Country: Costa Rica								
Hot week	3730	0.18	0	0.38	0	0	0	1
Threshold at 90th percentile	3730	25	24	3	22	22	29	30
Daily UTCI	3730	24	23	3.3	16	21	27	31
Agricultural employment	3730	12	12	0.52	11	12	13	13
Vulnerable employment	3730	20	20	0.59	20	20	21	21

Formality	0				Inf			-Inf
Wage labour	3730	76	76	0.39	76	76	77	77
GNI per capita (ln)	3730	9.3	9.3	0.039	9.2	9.3	9.4	9.4
Country: Croatia								
Hot week	3946	0.19	0	0.4	0	0	0	1
Threshold at 90th percentile	3946	25	25	1.7	20	24	26	28
Daily UTCI	3946	21	22	4.9	4.2	18	24	33
Agricultural employment	3946	8.3	7.6	1.1	7	7	9.2	9.5
Vulnerable employment	3946	9.3	8.7	1.2	7.5	7.5	10	10
Formality	0				Inf			-Inf
Wage labour	3946	86	86	1.4	84	84	88	88
GNI per capita (ln)	3946	9.5	9.5	0.025	9.4	9.4	9.5	9.5
Country: Cyprus								
Hot week	4924	0.16	0	0.36	0	0	0	1
Threshold at 90th percentile	4924	30	31	1.6	28	29	32	32
Daily UTCI	4924	25	24	4.4	11	21	27	34
Agricultural employment	2932	3.8	4	0.69	2.5	3.6	4.4	4.4
Vulnerable employment	2932	13	13	1.1	11	12	14	14
Formality	0				Inf			-Inf

Wage labour	2932	84	85	1.9	82	82	86	87
GNI per capita (ln)	2932	10	10	0.03	10	10	10	10
			Country: Czech Republic					
Hot week	4008	0.4	0	0.49	0	0	1	1
Threshold at 90th percentile	4008	21	21	1.4	18	20	22	25
Daily UTCI	4008	17	18	6	-5.1	14	22	30
Agricultural employment	4008	2.8	2.8	0.073	2.8	2.8	2.9	2.9
Vulnerable employment	4008	14	14	0.27	14	14	15	15
Formality	0				Inf			-Inf
Wage labour	4008	83	83	0.35	82	82	83	83
GNI per capita (ln)	4008	9.8	9.8	0.024	9.8	9.8	9.8	9.8
			Country: Denmark					
Hot week	4962	0.091	0	0.29	0	0	0	1
Threshold at 90th percentile	4962	16	16	1.6	11	15	17	18
Daily UTCI	4962	8.1	8.1	5.7	-12	4	12	23
Agricultural employment	4962	2.4	2.5	0.19	1.3	2.3	2.5	2.5
Vulnerable employment	4962	5.3	5.2	0.14	5.2	5.2	5.3	6.3
Formality	0				Inf			-Inf
Wage labour	4962	91	91	0.33	89	91	91	92

GNI per capita (ln)	4962	11	11	0.058	11	11	11	11
Country: Dominican Republic								
Hot week	3856	0.18	0	0.38	0	0	0	1
Threshold at 90th percentile	3856	29	30	1.1	23	29	30	32
Daily UTCI	3856	28	28	1.7	21	27	29	31
Agricultural employment	3856	9.9	9.6	1.3	8.9	8.9	9.7	31
Vulnerable employment	3856	41	41	1.6	40	40	41	73
Formality	3848	79	79	0	79	79	79	79
Wage labour	3856	56	56	1.5	26	56	57	57
GNI per capita (ln)	3856	8.8	8.8	0.082	7.3	8.8	8.9	8.9
Country: Ecuador								
Hot week	3943	0.0033	0	0.057	0	0	0	1
Threshold at 90th percentile	3943	23	29	7.4	10	17	30	31
Daily UTCI	3943	19	24	8.6	-1.7	14	26	29
Agricultural employment	3943	27	27	0.89	25	26	27	28
Vulnerable employment	3943	43	45	2.7	40	42	46	46
Formality	3943	88	88	0	88	88	88	88
Wage labour	3943	54	51	2.6	51	51	55	57
GNI per capita (ln)	3943	8.7	8.7	0.02	8.7	8.7	8.7	8.7

Country: Egypt

Hot week	3998	0.29	0	0.45	0	0	1	1
Threshold at 90th percentile	3998	30	30	1.7	26	30	31	36
Daily UTCI	3998	24	25	7	10	20	30	37
Agricultural employment	3998	26	26	0.94	25	26	27	28
Vulnerable employment	3998	23	25	2.5	20	20	25	25
Formality	3998	91	91	0	91	91	91	91
Wage labour	3998	65	63	3.7	61	61	69	70
GNI per capita (ln)	3998	8.1	8.1	0.047	8	8.1	8.1	8.1

Country: El Salvador

Hot week	3870	0.1	0	0.3	0	0	0	1
Threshold at 90th percentile	3870	29	28	1.7	27	28	30	33
Daily UTCI	3870	28	28	2.2	18	27	29	34
Agricultural employment	3870	19	19	0.25	18	19	19	19
Vulnerable employment	3870	36	36	0.35	35	35	36	36
Formality	3870	83	83	0	83	83	83	83
Wage labour	3870	60	60	0.32	60	60	60	60
GNI per capita (ln)	3870	8.2	8.2	0.022	8.1	8.1	8.2	8.2

Country: Estonia

Hot week	3985	0.39	0	0.49	0	0	1	1
Threshold at 90th percentile	3985	17	16	1.9	13	16	18	21
Daily UTCI	3985	13	13	5.8	-5	8.4	18	26
Agricultural employment	3985	3.9	3.9	0.55	3.5	3.9	3.9	7.9
Vulnerable employment	3985	5.7	5.7	0.36	5.5	5.7	5.8	8.6
Formality	0				Inf			-Inf
Wage labour	3985	90	90	0.56	87	90	91	91
GNI per capita (ln)	3985	9.8	9.8	0.033	9.6	9.8	9.8	9.9

Country: Ethiopia

Hot week	3823	0.3	0	0.46	0	0	1	1
Threshold at 90th percentile	3823	23	22	3.4	17	20	26	35
Daily UTCI	3823	22	22	3.1	15	20	24	34
Agricultural employment	3823	69	69	0.86	68	68	69	70
Vulnerable employment	3823	86	86	0.8	85	85	86	87
Formality	3823	96	96	0	96	96	96	96
Wage labour	3823	14	14	0.82	12	13	15	15
GNI per capita (ln)	3823	6.5	6.5	0.11	6.3	6.4	6.6	6.6

Country: Finland

Hot week	4969	0.2	0	0.4	0	0	0	1
Threshold at 90th percentile	4969	17	17	2	12	16	20	21
Daily UTCI	4969	8.5	8.7	6.6	-13	4	13	22
Agricultural employment	4969	4.1	4.2	0.26	1.8	3.9	4.3	4.3
Vulnerable employment	4969	9.7	9.8	0.36	6.2	9.8	10	10
Formality	0				Inf			-Inf
Wage labour	4969	86	86	0.45	86	86	86	90
GNI per capita (ln)	4969	11	11	0.043	11	11	11	11
				Country: France				
Hot week	3968	0.021	0	0.14	0	0	0	1
Threshold at 90th percentile	3968	21	21	2	16	19	23	25
Daily UTCI	3968	11	11	5.4	-4.7	6.4	15	24
Agricultural employment	3968	2.8	2.8	0.062	2.7	2.7	2.9	2.9
Vulnerable employment	3968	7.4	7.4	0.091	7.3	7.3	7.5	7.5
Formality	0				Inf			-Inf
Wage labour	3968	88	88	0.087	88	88	88	88
GNI per capita (ln)	3968	11	11	0.043	11	11	11	11
				Country: Gabon				
Hot week	3798	0	0	0	0	0	0	0

Threshold at 90th percentile	3798	30	30	1.2	27	29	31	32
Daily UTCI	3798	27	27	1.2	23	26	28	29
Agricultural employment	3798	32	32	0.59	31	31	32	33
Vulnerable employment	3798	29	29	0.26	29	29	29	30
Formality	0				Inf			-Inf
Wage labour	3798	68	68	0.24	68	68	68	68
GNI per capita (ln)	3798	8.9	8.9	0.13	8.8	8.8	9	9.1
Country: Georgia								
Hot week	3937	0.19	0	0.39	0	0	0	1
Threshold at 90th percentile	3937	23	24	2.5	17	22	25	28
Daily UTCI	3937	20	20	4.1	9	18	23	30
Agricultural employment	3937	42	44	6.9	19	43	44	46
Vulnerable employment	3937	50	52	6.8	28	50	52	55
Formality	0				Inf			-Inf
Wage labour	3937	48	47	5.9	44	46	48	68
GNI per capita (ln)	3937	8.5	8.4	0.29	8.3	8.3	8.5	9.4
Country: Germany								
Hot week	5953	0.066	0	0.25	0	0	0	1
Threshold at 90th percentile	5953	20	20	1.3	17	20	21	24

Daily UTCI	5953	7.5	8.3	8.2	-15	1.6	14	27
Agricultural employment	5953	1.4	1.4	0.061	1.3	1.3	1.4	1.4
Vulnerable employment	5953	6.1	6.1	0.18	5.8	6	6.3	6.3
Formality	0				Inf			-Inf
Wage labour	5953	89	89	0.29	89	89	90	90
GNI per capita (ln)	5953	11	11	0.035	11	11	11	11
			Country: Ghana					
Hot week	3622	0.029	0	0.17	0	0	0	1
Threshold at 90th percentile	3622	31	31	1.3	29	30	31	35
Daily UTCI	3622	29	29	2	24	28	30	35
Agricultural employment	3622	35	34	2.8	33	33	35	40
Vulnerable employment	3622	70	70	0.58	69	69	70	70
Formality	0				Inf			-Inf
Wage labour	3622	25	25	0.55	24	25	26	26
GNI per capita (ln)	3622	7.5	7.5	0.025	7.5	7.5	7.6	7.6
			Country: Greece					
Hot week	3933	0.19	0	0.4	0	0	0	1
Threshold at 90th percentile	3933	27	28	1.4	22	26	28	29
Daily UTCI	3933	23	23	3.8	11	20	26	33

Agricultural employment	3933	13	13	0.57	12	12	14	14
Vulnerable employment	3933	28	28	1.2	27	27	30	30
Formality	0				Inf			-Inf
Wage labour	3933	65	65	0.78	64	64	66	66
GNI per capita (ln)	3933	9.9	9.9	0.078	9.8	9.8	10	10
			Country: Guatemala					
Hot week	3768	0.23	0	0.42	0	0	0	1
Threshold at 90th percentile	3768	25	26	4.8	17	20	30	32
Daily UTCI	3768	24	25	4.9	14	19	28	34
Agricultural employment	3768	31	32	1	29	32	32	32
Vulnerable employment	3768	37	38	1.3	35	37	38	38
Formality	3768	86	86	0	86	86	86	86
Wage labour	3768	60	60	1.2	59	59	60	62
GNI per capita (ln)	3768	8.2	8.3	0.054	8.2	8.2	8.3	8.3
			Country: Guinea					
Hot week	3847	0.26	0	0.44	0	0	1	1
Threshold at 90th percentile	3847	30	30	1.2	27	29	31	31
Daily UTCI	3847	28	28	2	21	27	30	33
Agricultural employment	3847	63	64	0.97	62	63	65	65

Vulnerable employment	3847	91	92	0.32	91	91	92	92
Formality	3847	97	97	0	97	97	97	97
Wage labour	3847	7.4	7.2	0.41	6.9	6.9	7.6	8
GNI per capita (ln)	3847	6.7	6.6	0.038	6.6	6.6	6.7	6.7
Country: Haiti								
Hot week	1557	0.1	0	0.31	0	0	0	1
Threshold at 90th percentile	1557	31	31	0.86	28	30	31	33
Daily UTCI	1557	29	30	1.7	24	28	30	33
Agricultural employment	1557	30	30	0.47	30	30	31	31
Vulnerable employment	1557	73	73	0.12	73	73	73	73
Formality	0				Inf			-Inf
Wage labour	1557	26	26	0.12	26	26	26	26
GNI per capita (ln)	1557	7.2	7.2	0.027	7.2	7.2	7.3	7.3
Country: Honduras								
Hot week	3726	0.17	0	0.38	0	0	0	1
Threshold at 90th percentile	3726	28	27	2.4	23	26	30	32
Daily UTCI	3726	26	26	3	15	24	29	33
Agricultural employment	3726	29	29	1.7	27	29	32	32
Vulnerable employment	3726	40	39	1.8	35	38	41	42

Formality	3726	85	86	0.46	84	86	86	86
Wage labour	3726	50	50	2.9	48	49	52	62
GNI per capita (ln)	3726	7.7	7.6	0.12	7.5	7.6	7.7	8.3
				Country: Hong Kong				
Hot week	3856	0.057	0	0.23	0	0	0	1
Threshold at 90th percentile	3856	32	32	0.54	31	31	32	32
Daily UTCI	3856	25	28	8.1	-0.38	22	30	34
Agricultural employment	3856	0.19	0.19	0.0083	0.18	0.18	0.2	0.2
Vulnerable employment	3856	6	5.9	0.16	5.8	5.8	6.2	6.2
Formality	0				Inf			-Inf
Wage labour	3856	91	91	0.25	91	91	91	91
GNI per capita (ln)	3856	11	11	0.059	11	11	11	11
				Country: Hungary				
Hot week	3960	0.0061	0	0.078	0	0	0	1
Threshold at 90th percentile	3960	24	24	1.2	22	23	25	27
Daily UTCI	3960	12	16	11	-23	4.8	20	28
Agricultural employment	3960	4.9	4.9	0.24	2.9	4.7	5	5
Vulnerable employment	3960	5.8	5.8	0.71	5.7	5.7	5.8	12
Formality	0				Inf			-Inf

Wage labour	3960	89	89	0.57	85	89	90	90
GNI per capita (ln)	3960	9.5	9.5	0.033	9.5	9.5	9.5	9.8
				Country: Iceland				
Hot week	1616	0	0	0	0	0	0	0
Threshold at 90th percentile	1616	12	11	1.2	11	11	13	13
Daily UTCI	1616	-7.8	-7.6	6.4	-21	-12	-2.8	5.5
Agricultural employment	1616	3.9	3.9	0.19	3.8	3.8	4.2	4.2
Vulnerable employment	1616	8.5	8.3	0.42	8.1	8.1	9.2	9.2
Formality	0				Inf			-Inf
Wage labour	1616	88	88	0.28	87	87	88	88
GNI per capita (ln)	1616	11	11	0.071	11	11	11	11
				Country: India				
Hot week	11986	0.25	0	0.43	0	0	0	1
Threshold at 90th percentile	11986	33	34	2.2	21	33	34	36
Daily UTCI	11986	31	31	3.9	10	29	33	39
Agricultural employment	11986	45	45	0.74	44	45	46	46
Vulnerable employment	11986	77	77	0.9	75	76	78	78
Formality	11986	87	87	0	87	87	87	87
Wage labour	11986	22	21	0.81	20	20	22	23

GNI per capita (ln)	11986	7.4	7.4	0.059	7.4	7.4	7.4	7.5
				Country: Indonesia				
Hot week	3938	0.1	0	0.31	0	0	0	1
Threshold at 90th percentile	3938	29	28	2.7	24	26	31	32
Daily UTCI	3938	27	27	2.9	21	25	30	33
Agricultural employment	3938	32	32	1.3	31	31	33	34
Vulnerable employment	3938	48	48	0.91	47	48	48	50
Formality	3938	61	61	0	61	61	61	61
Wage labour	3938	48	49	0.95	47	49	49	49
GNI per capita (ln)	3938	8.2	8.1	0.025	8.1	8.1	8.2	8.2
				Country: Iran				
Hot week	4876	0.00062	0	0.025	0	0	0	1
Threshold at 90th percentile	4876	26	26	4.3	19	23	29	40
Daily UTCI	4876	14	15	8.9	-11	7.4	21	39
Agricultural employment	4876	18	18	0.16	18	18	18	18
Vulnerable employment	4876	41	40	0.55	40	40	41	41
Formality	0				Inf			-Inf
Wage labour	4876	56	56	0.57	55	55	56	56
GNI per capita (ln)	4876	8.7	8.6	0.088	8.6	8.6	8.8	8.8

Country: Iraq								
Hot week	4686	0	0	0	0	0	0	0
Threshold at 90th percentile	4686	38	38	2.7	30	36	40	42
Daily UTCI	4686	21	22	7.8	-0.2	16	27	38
Agricultural employment	4686	20	20	0.61	19	20	21	21
Vulnerable employment	4686	22	23	1.3	21	21	24	24
Formality	0				Inf			-Inf
Wage labour	4686	75	75	1.4	74	74	77	78
GNI per capita (ln)	4686	8.7	8.7	0.14	8.4	8.6	8.8	8.8
Country: Ireland								
Hot week	4892	0.055	0	0.23	0	0	0	1
Threshold at 90th percentile	4898	14	14	1.4	8.8	13	15	16
Daily UTCI	4898	4.4	5.2	7.1	-19	-0.32	10	20
Agricultural employment	4898	5.3	5.3	0.14	5.1	5.3	5.4	5.4
Vulnerable employment	4898	12	12	0.47	11	12	12	12
Formality	0				Inf			-Inf
Wage labour	4898	84	84	0.5	83	83	84	85
GNI per capita (ln)	4898	11	11	0.057	11	11	11	11

Country: Israel

Hot week	3996	0	0	0	0	0	0	0
Threshold at 90th percentile	3996	29	29	0.97	27	28	29	31
Daily UTCI	3996	21	21	4.7	6	18	24	30
Agricultural employment	3996	3	1	3.3	1	1	6.7	10
Vulnerable employment	3996	13	8	7.4	8	8	23	26
Formality	0				Inf			-Inf
Wage labour	3996	83	88	8.5	68	70	88	88
GNI per capita (ln)	3996	9.9	10	1	8.2	8.3	11	11

Country: Italy

Hot week	4896	0.0061	0	0.078	0	0	0	1
Threshold at 90th percentile	4896	25	25	2.5	13	24	27	30
Daily UTCI	4896	12	12	5.5	-8.9	8.5	16	26
Agricultural employment	4896	3.7	3.8	0.091	3.6	3.6	3.8	3.9
Vulnerable employment	4896	18	18	0.39	17	17	18	18
Formality	0				Inf			-Inf
Wage labour	4896	76	76	0.56	75	75	76	77
GNI per capita (ln)	4896	10	10	0.044	10	10	10	10

Country: Ivory Coast

Hot week	3733	0.34	0	0.48	0	0	1	1
Threshold at 90th percentile	3733	30	31	0.93	28	30	31	32
Daily UTCI	3733	30	30	1.6	24	29	31	33
Agricultural employment	3733	43	43	0.91	42	42	44	44
Vulnerable employment	3733	73	73	1.1	72	72	74	75
Formality	3733	88	88	0	88	88	88	88
Wage labour	3733	25	25	0.92	24	25	27	27
GNI per capita (ln)	3733	7.5	7.6	0.15	7.3	7.6	7.6	7.6
Country: Japan								
Hot week	4972	0.0026	0	0.051	0	0	0	1
Threshold at 90th percentile	4972	27	28	2.2	20	27	28	30
Daily UTCI	4972	15	16	6.6	-20	11	20	31
Agricultural employment	4972	3.6	3.6	0.1	3.4	3.5	3.7	3.7
Vulnerable employment	4972	8.9	9	0.35	8.4	8.6	9.3	9.3
Formality	0				Inf			-Inf
Wage labour	4972	89	89	0.41	89	89	89	90
GNI per capita (ln)	4972	11	11	0.065	11	11	11	11
Country: Jordan								
Hot week	3988	0.19	0	0.39	0	0	0	1

Threshold at 90th percentile	3988	26	26	1.4	24	26	28	30
Daily UTCI	3988	22	22	4.6	10	19	26	33
Agricultural employment	3988	2.8	2.8	0.11	2.7	2.7	2.9	3
Vulnerable employment	3988	10	10	0.21	9.8	10	10	10
Formality	0				Inf			-Inf
Wage labour	3988	86	86	0.034	86	86	86	86
GNI per capita (ln)	3988	8.3	8.3	0.015	8.3	8.3	8.3	8.3
Country: Kazakhstan								
Hot week	3988	0.48	0	0.5	0	0	1	1
Threshold at 90th percentile	3988	24	22	4.3	17	21	28	31
Daily UTCI	3988	22	22	6.4	0.091	17	26	35
Agricultural employment	3988	18	18	1.7	17	17	21	21
Vulnerable employment	3988	25	26	1.5	23	24	27	27
Formality	0				Inf			-Inf
Wage labour	3988	74	73	1.6	71	71	74	76
GNI per capita (ln)	3988	9.2	9.3	0.17	9	9.1	9.4	9.4
Country: Kenya								
Hot week	3843	0.34	0	0.47	0	0	1	1
Threshold at 90th percentile	3843	23	23	4.1	16	21	26	33

Daily UTCI	3843	22	22	4.4	11	19	24	34
Agricultural employment	3843	57	57	2.6	56	56	58	73
Vulnerable employment	3843	54	52	5.3	51	51	55	87
Formality	67	96	96	0	96	96	96	96
Wage labour	3843	46	48	5.6	12	45	49	49
GNI per capita (ln)	3843	7.2	7.3	0.15	6.3	7.1	7.3	7.3
				Country: Kosovo				
Hot week	3930	0.13	0	0.34	0	0	0	1
Threshold at 90th percentile	3930	23	24	1.7	19	23	24	26
Daily UTCI	3930	18	18	4	7.5	16	21	28
Agricultural employment	275	18	19	1.2	16	17	19	20
Vulnerable employment	275	26	27	2.9	19	26	28	28
Formality	0				Inf			-Inf
Wage labour	275	70	69	2.6	68	69	70	76
GNI per capita (ln)	3930	8.3	8.3	0.095	8.3	8.3	8.3	8.7
				Country: Kuwait				
Hot week	5001	0.012	0	0.11	0	0	0	1
Threshold at 90th percentile	5001	39	39	0.55	38	39	40	40
Daily UTCI	5001	27	30	8.4	-5.4	24	32	40

Agricultural employment	5001	2.1	2.1	0.075	1.9	2	2.1	2.2
Vulnerable employment	5001	0.76	0.68	0.25	0.5	0.5	1.1	1.1
Formality	0				Inf			-Inf
Wage labour	5001	99	99	0.29	98	98	99	99
GNI per capita (ln)	5001	11	11	0.17	10	10	11	11
Country: Kyrgyzstan								
Hot week	3960	0.63	1	0.48	0	0	1	1
Threshold at 90th percentile	3960	21	24	6.1	11	14	27	28
Daily UTCI	3960	21	23	6.4	4.7	14	26	31
Agricultural employment	3960	28	27	3.2	23	23	32	32
Vulnerable employment	3960	37	35	2.8	34	34	41	41
Formality	0				Inf			-Inf
Wage labour	3960	61	62	2.7	57	57	64	64
GNI per capita (ln)	3960	7.1	7	0.05	7	7	7.1	7.1
Country: Laos								
Hot week	827	0.1	0	0.31	0	0	0	1
Threshold at 90th percentile	827	30	29	2.2	25	28	32	32
Daily UTCI	827	28	29	2.5	22	26	31	32
Agricultural employment	827	58	64	12	31	64	64	64

Vulnerable employment	827	71	76	11	48	76	76	76
Formality	827	95	96	3.7	87	96	96	96
Wage labour	827	28	23	9.9	23	23	23	49
GNI per capita (ln)	827	7.9	7.7	0.37	7.7	7.7	7.7	8.7
Country: Latvia								
Hot week	3987	0.44	0	0.5	0	0	1	1
Threshold at 90th percentile	3987	18	19	1.5	17	17	20	22
Daily UTCI	3987	17	16	4.4	-4.3	14	19	27
Agricultural employment	3987	7.5	7.7	0.39	6.9	7.5	7.8	7.9
Vulnerable employment	3987	8.1	8.3	0.4	7.6	7.8	8.6	8.6
Formality	0				Inf			-Inf
Wage labour	3987	88	87	0.52	87	87	88	88
GNI per capita (ln)	3987	9.6	9.6	0.014	9.6	9.6	9.6	9.6
Country: Lebanon								
Hot week	4000	0.084	0	0.28	0	0	0	1
Threshold at 90th percentile	4000	26	27	1.8	22	25	28	28
Daily UTCI	4000	19	19	4.1	5.8	17	22	29
Agricultural employment	4000	13	13	0.39	12	12	13	13
Vulnerable employment	4000	28	28	0.75	27	27	28	29

Formality	0				Inf			-Inf
Wage labour	4000	64	64	0.33	64	64	64	64
GNI per capita (ln)	4000	8.9	8.9	0.017	8.9	8.9	8.9	8.9
				Country: Lesotho				
Hot week	1642	0.058	0	0.23	0	0	0	1
Threshold at 90th percentile	1642	20	21	1.8	15	19	21	22
Daily UTCI	1642	8.7	7.8	8.1	-8	1.9	17	21
Agricultural employment	1642	36	45	17	5.3	45	46	46
Vulnerable employment	1642	39	47	16	9.6	47	47	47
Formality	1260	91	91	0	91	91	91	91
Wage labour	1642	60	52	14	52	52	52	85
GNI per capita (ln)	1642	7.5	7.1	0.67	7.1	7.1	7.1	8.7
				Country: Liberia				
Hot week	1867	0.0043	0	0.065	0	0	0	1
Threshold at 90th percentile	1867	30	30	0.73	29	30	31	31
Daily UTCI	1867	29	29	1.1	26	28	30	32
Agricultural employment	1867	43	43	0.26	43	43	44	44
Vulnerable employment	1867	77	77	0.12	77	77	77	77
Formality	1867	71	71	0	71	71	71	71

Wage labour	1867	21	21	0.13	21	21	21	21
GNI per capita (ln)	1867	6.5	6.5	0	6.5	6.5	6.5	6.5
				Country: Libya				
Hot week	2966	0.031	0	0.17	0	0	0	1
Threshold at 90th percentile	2966	29	29	1.4	24	28	30	33
Daily UTCI	2966	22	23	4.9	7.8	18	27	36
Agricultural employment	2966	18	18	0.48	18	18	19	19
Vulnerable employment	2966	36	36	0.46	36	36	37	37
Formality	0				Inf			-Inf
Wage labour	2966	61	61	0.46	60	60	61	61
GNI per capita (ln)	2966	8.6	8.6	0.096	8.4	8.4	8.6	8.6
				Country: Lithuania				
Hot week	3932	0.78	1	0.42	0	1	1	1
Threshold at 90th percentile	3932	19	18	1.4	16	18	20	21
Daily UTCI	3932	21	20	3.9	6.2	18	24	27
Agricultural employment	3932	8.5	9.1	0.63	7.8	8	9.2	9.2
Vulnerable employment	3932	9.8	9.8	0.17	9.6	9.8	10	10
Formality	0				Inf			-Inf
Wage labour	3932	88	88	0.13	88	88	88	88

GNI per capita (ln)	3932	9.6	9.6	0.028	9.6	9.6	9.7	9.7
			Country: Luxembourg					
Hot week	4883	0.088	0	0.28	0	0	0	1
Threshold at 90th percentile	4883	18	19	1	17	18	19	20
Daily UTCI	4883	9.2	9.1	6.9	-9.1	4.5	14	26
Agricultural employment	4883	1.3	1.4	0.34	1	1	1.4	2.9
Vulnerable employment	4883	6.3	6.3	0.24	5.8	6.2	6.3	7.5
Formality	0				Inf			-Inf
Wage labour	4883	90	90	0.71	88	90	91	91
GNI per capita (ln)	4883	11	11	0.16	11	11	11	11
			Country: Macedonia					
Hot week	3810	0.28	0	0.45	0	0	1	1
Threshold at 90th percentile	3810	24	24	1.7	21	23	26	27
Daily UTCI	3810	20	21	4.5	8.5	17	23	33
Agricultural employment	3810	17	17	1.7	12	16	18	20
Vulnerable employment	3810	22	22	2.9	19	20	23	30
Formality	0				Inf			-Inf
Wage labour	3810	73	74	3.3	64	73	76	76
GNI per capita (ln)	3810	8.7	8.5	0.4	8.5	8.5	8.6	10

Country: Madagascar

Hot week	3997	0.14	0	0.35	0	0	0	1
Threshold at 90th percentile	3997	26	26	3.2	22	23	29	32
Daily UTCI	3997	22	22	4.8	8.6	19	26	32
Agricultural employment	3997	66	66	0.73	65	66	67	67
Vulnerable employment	3997	85	86	0.33	85	85	86	86
Formality	0				Inf			-Inf
Wage labour	3997	11	11	0.16	11	11	11	11
GNI per capita (ln)	3997	6.2	6.2	0.041	6.2	6.2	6.3	6.3

Country: Malawi

Hot week	3909	0	0	0	0	0	0	0
Threshold at 90th percentile	3909	26	25	2.2	22	24	28	32
Daily UTCI	3909	20	20	3.5	9.7	17	22	29
Agricultural employment	3909	77	77	2.5	52	77	77	77
Vulnerable employment	3909	62	60	5.5	60	60	61	84
Formality	3656	90	90	0	90	90	90	90
Wage labour	3909	37	38	5.7	13	38	39	39
GNI per capita (ln)	3909	5.9	5.9	0.18	5.8	5.9	5.9	7.5

Country: Malaysia

Hot week	2959	0.044	0	0.2	0	0	0	1
Threshold at 90th percentile	2959	32	32	0.67	30	31	32	33
Daily UTCI	2959	30	30	1.2	23	29	31	34
Agricultural employment	2959	13	13	0.057	12	12	13	13
Vulnerable employment	2959	21	21	0.55	21	21	22	22
Formality	2959	71	71	0	71	71	71	71
Wage labour	2959	75	75	0.72	74	74	75	75
GNI per capita (ln)	2959	9.3	9.3	0.02	9.3	9.3	9.3	9.3

Country: Mali

Hot week	3692	0.18	0	0.39	0	0	0	1
Threshold at 90th percentile	3692	33	33	1.3	31	32	34	37
Daily UTCI	3692	31	30	2.3	24	29	32	37
Agricultural employment	3692	64	63	1.7	62	63	65	67
Vulnerable employment	3692	81	80	0.97	80	80	82	82
Formality	3692	86	86	0	86	86	86	86
Wage labour	3692	18	19	0.85	18	18	20	20
GNI per capita (ln)	3692	6.7	6.7	0.02	6.6	6.6	6.7	6.7

Country: Malta

Hot week	5015	0.18	0	0.39	0	0	0	1
Threshold at 90th percentile	5015	27	26	0.9	26	26	27	28
Daily UTCI	5015	15	15	8.7	-1.7	7.5	24	28
Agricultural employment	5015	3.7	3.8	0.091	3.6	3.6	3.8	3.9
Vulnerable employment	5015	18	18	0.39	17	17	18	18
Formality	0				Inf			-Inf
Wage labour	5015	76	76	0.56	75	75	76	77
GNI per capita (ln)	5015	10	10	0.044	10	10	10	10
			Country: Mauritania					
Hot week	3820	0	0	0	0	0	0	0
Threshold at 90th percentile	3820	33	35	3.7	26	29	36	37
Daily UTCI	3820	24	24	6.1	12	18	29	36
Agricultural employment	3820	33	33	0.6	32	32	34	34
Vulnerable employment	3820	54	54	0.41	53	53	54	54
Formality	3820	86	86	0	86	86	86	86
Wage labour	3820	43	43	0.2	42	42	43	43
GNI per capita (ln)	3820	7.4	7.4	0.059	7.3	7.3	7.5	7.5
			Country: Mauritius					
Hot week	2984	0	0	0	0	0	0	0

Threshold at 90th percentile	2984	29	29	0.96	27	29	29	30
Daily UTCI	2984	22	23	3.7	14	19	25	29
Agricultural employment	2984	6.7	6.7	0.3	6.4	6.4	7.2	7.2
Vulnerable employment	2984	16	16	0.074	16	16	16	16
Formality	0				Inf			-Inf
Wage labour	2984	79	79	0.048	79	79	79	79
GNI per capita (ln)	2984	9.3	9.3	0.013	9.3	9.3	9.3	9.3
				Country: Mexico				
Hot week	3894	0.4	0	0.49	0	0	1	1
Threshold at 90th percentile	3894	24	22	5.3	17	20	29	35
Daily UTCI	3894	23	21	5.3	11	18	27	35
Agricultural employment	3894	13	13	0.27	13	13	13	14
Vulnerable employment	3894	27	28	0.43	27	27	28	28
Formality	0				Inf			-Inf
Wage labour	3894	68	68	0.3	68	68	68	69
GNI per capita (ln)	3894	9.2	9.2	0.064	9.1	9.1	9.2	9.3
				Country: Moldova				
Hot week	3939	0.85	1	0.36	0	1	1	1
Threshold at 90th percentile	3939	24	24	0.84	22	24	25	26

Daily UTCI	3939	25	26	3.4	13	23	27	32
Agricultural employment	3939	32	32	2.7	15	30	34	34
Vulnerable employment	3939	34	34	3	14	32	36	36
Formality	0				Inf			-Inf
Wage labour	3939	65	65	2.9	63	63	67	84
GNI per capita (ln)	3939	8.2	8.1	0.21	7.8	8.1	8.2	9.2

Country: Mongolia

Hot week	3975	0.25	0	0.43	0	0	1	1
Threshold at 90th percentile	3975	18	18	2.3	13	16	19	25
Daily UTCI	3975	14	15	5.9	-0.21	10	19	28
Agricultural employment	3975	29	28	0.89	28	28	29	30
Vulnerable employment	3975	49	49	1	47	49	49	49
Formality	0				Inf			-Inf
Wage labour	3975	50	50	0.92	49	49	50	52
GNI per capita (ln)	3975	8.2	8.2	0.098	8.1	8.2	8.3	8.3

Country: Montenegro

Hot week	3955	0.23	0	0.42	0	0	0	1
Threshold at 90th percentile	3955	22	22	2.9	16	20	24	29
Daily UTCI	3955	18	18	5.2	1.7	15	21	32

Agricultural employment	3829	7.7	7.7	3.8	5.7	7.7	7.9	42
Vulnerable employment	3829	13	13	4.9	11	12	13	56
Formality	0				Inf			-Inf
Wage labour	3829	79	79	4.3	41	78	79	81
GNI per capita (ln)	3955	8.9	8.9	0.12	8.3	8.9	8.9	8.9
Country: Morocco								
Hot week	3832	0	0	0	0	0	0	0
Threshold at 90th percentile	3832	27	28	2.6	19	26	29	34
Daily UTCI	3832	14	15	6.1	-3	10	19	28
Agricultural employment	3832	36	37	0.67	35	36	37	37
Vulnerable employment	3832	49	49	0.66	48	49	49	49
Formality	0				Inf			-Inf
Wage labour	3832	49	48	0.67	48	48	49	50
GNI per capita (ln)	3832	8	8	0.024	8	8	8	8
Country: Mozambique								
Hot week	1642	0.032	0	0.18	0	0	0	1
Threshold at 90th percentile	1642	29	29	1.9	23	28	30	32
Daily UTCI	1642	22	21	4.4	11	19	24	35
Agricultural employment	1642	72	71	0.53	71	71	72	72

Vulnerable employment	1642	83	82	0.77	82	82	84	84
Formality	0				Inf			-Inf
Wage labour	1642	15	16	0.75	14	14	16	16
GNI per capita (ln)	1642	6.3	6.2	0.15	6.2	6.2	6.5	6.5
			Country: Myanmar					
Hot week	4651	0.16	0	0.37	0	0	0	1
Threshold at 90th percentile	4651	32	32	1.7	28	32	33	34
Daily UTCI	4651	29	29	3.3	18	27	31	36
Agricultural employment	4651	51	51	0.61	51	51	52	52
Vulnerable employment	4651	61	61	0.86	60	60	62	63
Formality	4651	84	84	3.1	79	84	84	88
Wage labour	4651	36	36	0.87	34	35	37	37
GNI per capita (ln)	4651	7.1	7.1	0.013	7.1	7.1	7.1	7.1
			Country: Namibia					
Hot week	1770	0.099	0	0.3	0	0	0	1
Threshold at 90th percentile	1770	26	27	2.1	21	26	27	28
Daily UTCI	1770	18	17	5.5	3.9	14	24	29
Agricultural employment	1770	25	21	4	21	21	29	29
Vulnerable employment	1770	29	28	1	28	28	30	30

Formality	1770	84	84	0	84	84	84	84
Wage labour	1770	66	67	0.2	66	66	67	67
GNI per capita (ln)	1770	8.5	8.4	0.11	8.4	8.4	8.7	8.7
				Country: Nepal				
Hot week	3977	0.19	0	0.39	0	0	0	1
Threshold at 90th percentile	3977	27	27	5.1	12	25	32	33
Daily UTCI	3977	25	26	5.9	5.1	22	30	35
Agricultural employment	3977	65	67	6.4	44	66	67	67
Vulnerable employment	3977	78	78	0.69	75	78	78	79
Formality	368	87	87	0	87	87	87	87
Wage labour	3977	21	21	0.54	20	20	21	23
GNI per capita (ln)	3977	6.9	6.8	0.18	6.8	6.8	6.9	7.5
				Country: Netherlands				
Hot week	4979	0.081	0	0.27	0	0	0	1
Threshold at 90th percentile	4979	18	18	1	14	17	19	20
Daily UTCI	4979	10	12	6.8	-16	5.6	15	24
Agricultural employment	4979	2.3	2.3	0.021	2.3	2.3	2.3	2.3
Vulnerable employment	4979	13	13	0.13	13	13	13	13
Formality	0				Inf			-Inf

Wage labour	4979	83	83	0.097	83	83	83	83
GNI per capita (ln)	4979	11	11	0.045	11	11	11	11
				Country: New Zealand				
Hot week	4996	0.028	0	0.17	0	0	0	1
Threshold at 90th percentile	4996	17	18	2.4	11	16	19	20
Daily UTCI	4996	6.6	6.6	6.5	-18	2	11	23
Agricultural employment	4996	6.3	6.2	0.16	6.1	6.2	6.3	6.6
Vulnerable employment	4996	12	12	0.47	11	12	12	12
Formality	0				Inf			-Inf
Wage labour	4996	84	85	1.6	81	82	85	85
GNI per capita (ln)	4996	11	11	0.028	11	11	11	11
				Country: Nicaragua				
Hot week	3812	0.36	0	0.48	0	0	1	1
Threshold at 90th percentile	3812	29	29	2.3	24	27	30	34
Daily UTCI	3812	28	29	3	19	26	30	35
Agricultural employment	3812	31	30	0.58	30	30	31	31
Vulnerable employment	3812	39	38	1.1	37	37	40	40
Formality	3812	84	84	0	84	84	84	84
Wage labour	3812	56	56	1	54	54	57	57

GNI per capita (ln)	3812	7.6	7.6	0.039	7.5	7.5	7.6	7.6
				Country: Niger				
Hot week	3725	0.31	0	0.46	0	0	1	1
Threshold at 90th percentile	3725	34	34	0.92	32	33	34	37
Daily UTCI	3725	32	32	2.2	25	31	34	38
Agricultural employment	3725	74	74	0.28	73	73	74	74
Vulnerable employment	3725	94	94	0.12	94	94	94	94
Formality	3725	86	86	0	86	86	86	86
Wage labour	3725	5.2	5.2	0.13	5	5	5.3	5.3
GNI per capita (ln)	3725	6.3	6.3	0.05	6.3	6.3	6.4	6.4
				Country: Nigeria				
Hot week	3728	0.27	0	0.44	0	0	1	1
Threshold at 90th percentile	3728	31	31	1.2	28	31	32	36
Daily UTCI	3728	30	30	2.3	23	28	31	37
Agricultural employment	3728	37	37	0.53	36	36	37	38
Vulnerable employment	3728	82	82	0.46	81	81	82	82
Formality	3728	58	58	0	58	58	58	58
Wage labour	3728	18	18	0.46	17	17	19	19
GNI per capita (ln)	3728	7.8	7.8	0.13	7.6	7.6	7.9	8

Country: Northern Cyprus

Hot week	3998	0.08	0	0.27	0	0	0	1
Threshold at 90th percentile	3998	31	31	1.2	28	30	32	32
Daily UTCI	3998	20	20	5.9	4.5	16	25	34
Agricultural employment	845	4.1	4.4	0.33	3.6	3.6	4.4	4.4
Vulnerable employment	845	13	14	0.82	12	12	14	14
Formality	0				Inf			-Inf
Wage labour	845	84	82	1.7	82	82	86	86
GNI per capita (ln)	845	10	10	0.029	10	10	10	10

Country: Norway

Hot week	5975	0.053	0	0.22	0	0	0	1
Threshold at 90th percentile	5982	15	15	2.4	6.7	14	16	18
Daily UTCI	5982	4	3.9	6.3	-24	-0.18	8.9	18
Agricultural employment	5982	2.1	2.1	0.092	2	2.1	2.2	2.2
Vulnerable employment	5982	5.2	5.1	0.2	4.7	5.1	5.3	5.3
Formality	0				Inf			-Inf
Wage labour	5982	93	93	0.23	93	93	93	93
GNI per capita (ln)	5982	11	11	0.13	11	11	12	12

Country: Pakistan

Hot week	4509	0.084	0	0.28	0	0	0	1
Threshold at 90th percentile	4509	34	36	4.8	23	35	37	37
Daily UTCI	4509	27	29	6.8	5	25	32	37
Agricultural employment	4509	41	41	1	40	40	42	42
Vulnerable employment	4509	59	59	0.83	58	58	59	60
Formality	0				Inf			-Inf
Wage labour	4509	40	40	0.81	39	39	41	41
GNI per capita (ln)	4509	7.2	7.2	0.052	7.1	7.1	7.2	7.2

Country: Palestinian Territories

Hot week	3871	0	0	0	0	0	0	0
Threshold at 90th percentile	3871	29	29	0.7	28	29	30	31
Daily UTCI	3871	23	23	2.9	16	21	24	31
Agricultural employment	3871	14	8.7	8.3	6.7	7.4	25	28
Vulnerable employment	3871	24	25	1.8	20	23	25	26
Formality	1208	91	91	0	91	91	91	91
Wage labour	3871	68	69	2.8	61	68	69	70
GNI per capita (ln)	3871	8.2	8.2	0.079	8	8.1	8.3	8.3

Country: Panama

Hot week	2769	0.12	0	0.32	0	0	0	1
Threshold at 90th percentile	2769	28	27	1.6	27	27	29	32
Daily UTCI	2769	27	26	2.4	22	25	28	33
Agricultural employment	2769	15	15	0.43	15	15	16	16
Vulnerable employment	2769	31	31	0.97	30	30	32	32
Formality	0				Inf			-Inf
Wage labour	2769	66	66	1.3	64	64	67	67
GNI per capita (ln)	2769	9.4	9.4	0.055	9.3	9.3	9.5	9.5
				Country: Paraguay				
Hot week	3255	0.086	0	0.28	0	0	0	1
Threshold at 90th percentile	3255	30	31	0.71	29	30	31	33
Daily UTCI	3255	21	21	7.1	3.4	15	26	33
Agricultural employment	3255	21	21	0.97	20	20	22	22
Vulnerable employment	3255	38	39	0.78	37	37	39	39
Formality	3255	96	96	0	96	96	96	96
Wage labour	3255	56	56	0.23	56	56	56	56
GNI per capita (ln)	3255	8.6	8.6	0.032	8.6	8.6	8.7	8.7
				Country: Peru				
Hot week	3944	0	0	0	0	0	0	0

Threshold at 90th percentile	3944	22	25	6.9	7.2	15	27	32
Daily UTCI	3944	15	16	6.4	-1.8	11	18	31
Agricultural employment	3944	28	28	0.26	28	28	28	28
Vulnerable employment	3944	51	51	0.41	50	50	51	51
Formality	3944	89	89	0	89	89	89	89
Wage labour	3944	45	45	0.37	45	45	45	46
GNI per capita (ln)	3944	8.7	8.8	0.03	8.7	8.7	8.8	8.8
				Country: Philippines				
Hot week	1990	0.021	0	0.14	0	0	0	1
Threshold at 90th percentile	1990	31	31	2.2	24	30	33	33
Daily UTCI	1990	29	29	2.3	21	28	31	33
Agricultural employment	1990	26	25	0.73	25	25	27	27
Vulnerable employment	1990	35	34	0.62	34	34	35	35
Formality	1990	93	93	0	93	93	93	93
Wage labour	1990	62	62	0.42	61	61	62	62
GNI per capita (ln)	1990	8.2	8.2	0.011	8.1	8.1	8.2	8.2
				Country: Poland				
Hot week	3976	0.25	0	0.43	0	0	1	1
Threshold at 90th percentile	3976	20	20	1.4	15	19	21	23

Daily UTCI	3976	15	14	5.9	-4.9	11	19	27
Agricultural employment	3976	11	11	0.57	10	10	11	12
Vulnerable employment	3976	17	17	0.37	16	16	17	17
Formality	0				Inf			-Inf
Wage labour	3976	79	79	0.38	79	79	80	80
GNI per capita (ln)	3976	9.5	9.5	0.028	9.5	9.5	9.5	9.5
				Country: Portugal				
Hot week	4996	0.1	0	0.3	0	0	0	1
Threshold at 90th percentile	4996	21	22	2.2	17	20	23	29
Daily UTCI	4996	13	14	6.5	-9.6	9	17	30
Agricultural employment	4996	7.6	7.5	0.91	6.4	6.9	8.6	8.6
Vulnerable employment	4996	14	14	0.89	12	13	15	15
Formality	0				Inf			-Inf
Wage labour	4996	81	82	1.1	80	80	82	83
GNI per capita (ln)	4996	9.9	9.9	0.026	9.9	9.9	10	10
				Country: Puerto Rico				
Hot week	468	0	0	0	0	0	0	0
Threshold at 90th percentile	468	28	28	0.48	28	28	28	29
Daily UTCI	468	23	23	0.82	21	23	24	26

Agricultural employment	468	1.2	1.2	0	1.2	1.2	1.2	1.2
Vulnerable employment	468	9.3	9.3	0	9.3	9.3	9.3	9.3
Formality	0				Inf			-Inf
Wage labour	468	84	84	0	84	84	84	84
GNI per capita (ln)	468	9.9	9.9	0	9.9	9.9	9.9	9.9
				Country: Qatar				
Hot week	965	0	0	0	0	0	0	0
Threshold at 90th percentile	965	40	40	0.29	39	40	40	40
Daily UTCI	965	18	17	3.7	2.6	15	21	27
Agricultural employment	965	1.2	1.2	0	1.2	1.2	1.2	1.2
Vulnerable employment	965	0.14	0.14	0	0.14	0.14	0.14	0.14
Formality	0				Inf			-Inf
Wage labour	965	100	100	0	100	100	100	100
GNI per capita (ln)	965	11	11	0	11	11	11	11
				Country: Romania				
Hot week	3941	0.15	0	0.36	0	0	0	1
Threshold at 90th percentile	3941	24	24	2	18	22	25	28
Daily UTCI	3941	17	17	6.2	-8.1	14	22	28
Agricultural employment	3941	25	23	2.8	15	23	26	28

Vulnerable employment	3941	27	26	3.1	14	25	28	31
Formality	0				Inf			-Inf
Wage labour	3941	72	73	3.1	68	71	74	84
GNI per capita (ln)	3941	9.1	9.2	0.22	7.8	9.2	9.2	9.2
			Country: Russia					
Hot week	7951	0.14	0	0.35	0	0	0	1
Threshold at 90th percentile	7951	21	21	2.7	16	18	22	27
Daily UTCI	7951	13	14	7.3	-17	8.3	18	31
Agricultural employment	7951	6.6	6.7	0.93	5.9	6.7	6.7	16
Vulnerable employment	7951	5.9	5.9	0.91	5.3	5.9	6.2	15
Formality	0				Inf			-Inf
Wage labour	7951	93	93	0.87	84	93	93	93
GNI per capita (ln)	7951	9.3	9.2	0.23	7.8	9.1	9.4	9.6
			Country: Rwanda					
Hot week	3750	0.019	0	0.14	0	0	0	1
Threshold at 90th percentile	3750	21	21	1.7	18	20	22	25
Daily UTCI	3750	20	19	1.9	14	18	21	24
Agricultural employment	3750	66	66	1.4	65	65	67	68
Vulnerable employment	3750	68	68	0.58	68	68	68	69

Formality	0				Inf			-Inf
Wage labour	3750	32	32	0.58	31	32	32	32
GNI per capita (ln)	3750	6.6	6.6	0.0095	6.6	6.6	6.6	6.6
			Country: Saudi Arabia					
Hot week	5038	0.044	0	0.21	0	0	0	1
Threshold at 90th percentile	5038	35	35	2.8	25	34	36	39
Daily UTCI	5038	29	32	7.4	6.9	26	34	39
Agricultural employment	5038	5	5.3	0.83	3.6	5	5.3	6.1
Vulnerable employment	5038	3	3	0.054	2.9	2.9	3	3
Formality	0				Inf			-Inf
Wage labour	5038	95	95	0.15	95	95	95	95
GNI per capita (ln)	5038	10	10	0.096	9.9	10	10	10
			Country: Senegal					
Hot week	3853	0.019	0	0.14	0	0	0	1
Threshold at 90th percentile	3853	31	31	1.6	28	30	32	36
Daily UTCI	3853	24	25	5.9	12	19	28	35
Agricultural employment	3853	33	33	1	32	32	34	34
Vulnerable employment	3853	65	65	2.5	53	64	66	67
Formality	3853	88	88	0.42	86	88	88	88

Wage labour	3853	34	34	2	32	33	35	43
GNI per capita (ln)	3853	7.2	7.2	0.052	7.1	7.2	7.2	7.5
Country: Serbia								
Hot week	3938	0.2	0	0.4	0	0	0	1
Threshold at 90th percentile	3938	25	25	1.9	19	24	27	28
Daily UTCI	3938	19	19	4.9	4.6	16	23	31
Agricultural employment	3938	19	19	1	17	18	20	20
Vulnerable employment	3938	27	28	0.7	26	27	28	28
Formality	0				Inf			-Inf
Wage labour	3938	69	69	0.61	68	68	69	70
GNI per capita (ln)	3938	8.7	8.6	0.044	8.6	8.6	8.7	8.7
Country: Sierra Leone								
Hot week	1689	0.81	1	0.39	0	1	1	1
Threshold at 90th percentile	1689	31	31	0.53	30	31	31	32
Daily UTCI	1689	31	31	0.87	29	31	32	33
Agricultural employment	1689	57	56	0.44	56	56	57	57
Vulnerable employment	1689	88	88	0.005	88	88	88	88
Formality	1689	91	91	0	91	91	91	91
Wage labour	1689	9.5	9.4	0.07	9.4	9.4	9.6	9.6

GNI per capita (ln)	1689	6.2	6.3	0.03	6.2	6.2	6.3	6.3
				Country: Slovakia				
Hot week	3994	0.041	0	0.2	0	0	0	1
Threshold at 90th percentile	3994	22	21	1.9	19	20	23	25
Daily UTCI	3994	15	15	4.6	0.17	12	18	28
Agricultural employment	3994	3.1	3.2	0.3	2.7	2.9	3.5	3.5
Vulnerable employment	3994	12	12	0.093	12	12	12	12
Formality	0				Inf			-Inf
Wage labour	3994	85	85	0.12	85	85	85	85
GNI per capita (ln)	3994	9.8	9.8	0.039	9.7	9.7	9.8	9.8
				Country: Slovenia				
Hot week	4727	0	0	0	0	0	0	0
Threshold at 90th percentile	4727	23	23	1.7	19	21	24	27
Daily UTCI	4727	11	12	4.9	-14	9.3	14	22
Agricultural employment	4727	7.6	7.1	1.9	5	5.6	9.6	9.6
Vulnerable employment	4727	12	13	2.3	7.5	11	15	15
Formality	0				Inf			-Inf
Wage labour	4727	84	84	1.9	81	81	85	88
GNI per capita (ln)	4727	9.9	10	0.21	9.4	10	10	10

Country: Somalia

Hot week	2805	0.16	0	0.37	0	0	0	1
Threshold at 90th percentile	2805	29	30	3.3	23	27	31	35
Daily UTCI	2805	26	27	3.9	15	24	29	34
Agricultural employment	2805	81	81	0.18	81	81	81	81
Vulnerable employment	2805	91	91	0.061	91	91	91	91
Formality	0				Inf			-Inf
Wage labour	2805	8.2	8.2	0.044	8.1	8.1	8.2	8.2
GNI per capita (ln)	2805	6	6	0.0076	6	6	6	6

Country: South Africa

Hot week	3899	0	0	0	0	0	0	0
Threshold at 90th percentile	3899	23	23	1.6	20	22	24	28
Daily UTCI	3899	11	11	5.2	-3.5	7.4	14	25
Agricultural employment	3899	5.3	5.6	0.38	4.7	5.3	5.6	5.6
Vulnerable employment	3899	9.4	9.6	0.4	8.8	9.4	9.9	9.9
Formality	0				Inf			-Inf
Wage labour	3899	85	85	0.51	85	85	85	86
GNI per capita (ln)	3899	8.8	8.7	0.089	8.7	8.7	8.8	8.9

Country: South Korea

Hot week	4927	0.058	0	0.23	0	0	0	1
Threshold at 90th percentile	4927	27	26	1.5	22	26	28	30
Daily UTCI	4927	16	17	7.9	-15	10	22	30
Agricultural employment	4927	5.2	5.2	0.29	4.9	4.9	5.6	5.6
Vulnerable employment	4927	20	20	0.61	19	19	21	21
Formality	0				Inf			-Inf
Wage labour	4927	74	74	0.65	73	73	75	75
GNI per capita (ln)	4927	10	10	0.028	10	10	10	10

Country: South Sudan

Hot week	2896	0.011	0	0.1	0	0	0	1
Threshold at 90th percentile	2896	32	32	1.3	30	31	33	35
Daily UTCI	2896	29	29	2	20	28	30	33
Agricultural employment	2896	62	62	0.29	61	62	62	62
Vulnerable employment	2896	90	90	0.57	89	90	91	91
Formality	2896	80	80	0	80	80	80	80
Wage labour	2896	8.6	8.3	0.46	8	8	9	9.1
GNI per capita (ln)	2896	7.1	7.1	0.046	7	7.1	7.1	7.1

Country: Spain

Hot week	4915	0.023	0	0.15	0	0	0	1
Threshold at 90th percentile	4915	25	25	3.2	19	23	27	31
Daily UTCI	4915	11	11	6.7	-13	6.3	16	29
Agricultural employment	4915	4.3	4.2	1.7	4.1	4.2	4.2	38
Vulnerable employment	4915	12	12	2	11	12	13	50
Formality	0				Inf			-Inf
Wage labour	4915	83	83	1.9	47	82	83	84
GNI per capita (ln)	4915	10	10	0.12	8	10	10	10
Country: Sri Lanka								
Hot week	3172	0.14	0	0.35	0	0	0	1
Threshold at 90th percentile	3172	30	31	1.9	25	30	31	32
Daily UTCI	3172	28	28	2.6	19	27	30	34
Agricultural employment	3172	28	29	1.3	26	26	29	29
Vulnerable employment	3172	40	41	0.7	39	39	41	41
Formality	0				Inf			-Inf
Wage labour	3172	57	56	0.67	56	56	58	58
GNI per capita (ln)	3172	8.2	8.2	0.025	8.2	8.2	8.3	8.3
Country: Sudan								
Hot week	923	0	0	0	0	0	0	0

Threshold at 90th percentile	923	33	34	1	32	33	34	36
Daily UTCI	923	22	23	4.7	9	19	27	29
Agricultural employment	923	43	42	6.5	28	42	42	61
Vulnerable employment	923	54	53	12	25	53	53	89
Formality	923	95	97	4.9	80	97	97	97
Wage labour	923	42	44	11	9.1	44	44	61
GNI per capita (ln)	923	7.1	7	0.23	7	7	7	8.1
				Country: Sweden				
Hot week	4964	0.096	0	0.3	0	0	0	1
Threshold at 90th percentile	4964	16	16	1.5	12	15	18	19
Daily UTCI	4964	8.2	8.4	5.8	-11	4.4	13	22
Agricultural employment	4964	1.9	2	0.074	1.8	1.9	2	2
Vulnerable employment	4964	6.5	6.7	0.2	6.2	6.4	6.7	6.7
Formality	0				Inf			-Inf
Wage labour	4964	90	90	0.19	90	90	90	90
GNI per capita (ln)	4964	11	11	0.064	11	11	11	11
				Country: Switzerland				
Hot week	4476	0.12	0	0.33	0	0	0	1
Threshold at 90th percentile	4476	21	21	2.3	12	20	23	25

Daily UTCI	4476	14	14	6.2	-5.3	9.4	19	28
Agricultural employment	4476	3.4	3.4	0.31	1.3	3.4	3.6	3.6
Vulnerable employment	4476	9.1	9.1	0.45	5.8	9	9.3	9.3
Formality	0				Inf			-Inf
Wage labour	4476	85	85	0.75	85	85	85	90
GNI per capita (ln)	4476	11	11	0.14	11	11	11	11
				Country: Syria				
Hot week	949	0	0	0	0	0	0	0
Threshold at 90th percentile	949	29	29	2	25	28	30	34
Daily UTCI	949	1.9	3.2	5.7	-8.3	-3	6.9	12
Agricultural employment	949	12	12	0	12	12	12	12
Vulnerable employment	949	38	38	0	38	38	38	38
Formality	0				Inf			-Inf
Wage labour	949	59	59	0	59	59	59	59
GNI per capita (ln)	949	6.9	6.9	0	6.9	6.9	6.9	6.9
				Country: Taiwan				
Hot week	4890	0.062	0	0.24	0	0	0	1
Threshold at 90th percentile	4890	31	31	1.4	25	30	32	33
Daily UTCI	4890	26	27	4.1	9	23	29	34

Agricultural employment	0				Inf			-Inf
Vulnerable employment	0				Inf			-Inf
Formality	0				Inf			-Inf
Wage labour	0				Inf			-Inf
GNI per capita (ln)	0				Inf			-Inf
Country: Tajikistan								
Hot week	3015	0.48	0	0.5	0	0	1	1
Threshold at 90th percentile	3015	23	27	7	7.6	15	29	30
Daily UTCI	3015	23	25	7.1	4.9	16	29	35
Agricultural employment	3015	48	49	1	47	48	49	49
Vulnerable employment	3015	34	35	1.4	32	34	36	36
Formality	0				Inf			-Inf
Wage labour	3015	66	65	1.4	64	64	66	68
GNI per capita (ln)	3015	7.1	7.1	0.11	6.9	7	7.2	7.2
Country: Thailand								
Hot week	3997	0.32	0	0.46	0	0	1	1
Threshold at 90th percentile	3997	33	33	0.97	29	32	33	36
Daily UTCI	3997	31	31	2	23	30	33	37
Agricultural employment	3997	33	32	4.4	31	31	33	66

Vulnerable employment	3997	49	49	3.9	48	48	50	78
Formality	3997	87	87	1.3	87	87	87	96
Wage labour	3997	48	48	3.6	21	47	49	49
GNI per capita (ln)	3997	8.6	8.6	0.14	7.6	8.6	8.7	8.7
Country: The Gambia								
Hot week	1000	0	0	0	0	0	0	0
Threshold at 90th percentile	1000	31	32	1.5	30	30	33	34
Daily UTCI	1000	19	20	2.1	15	17	21	25
Agricultural employment	1000	29	28	1	28	28	28	32
Vulnerable employment	1000	70	71	2.2	64	71	71	71
Formality	112	88	88	0	88	88	88	88
Wage labour	1000	28	27	2.4	27	27	27	35
GNI per capita (ln)	1000	6.6	6.5	0.21	6.5	6.5	6.5	7.2
Country: Togo								
Hot week	1950	0.075	0	0.26	0	0	0	1
Threshold at 90th percentile	1950	32	32	0.96	29	31	32	34
Daily UTCI	1950	30	30	2	24	28	31	35
Agricultural employment	1950	35	36	1.6	34	34	36	41
Vulnerable employment	1950	77	77	2.2	76	76	77	88

Formality	1950	81	81	1.8	81	81	81	91
Wage labour	1950	21	21	2.1	11	21	22	22
GNI per capita (ln)	1950	6.6	6.6	0.094	6.5	6.5	6.6	7

Country: Trinidad & Tobago

Hot week	495	0.15	0	0.36	0	0	0	1
Threshold at 90th percentile	495	29	30	1.3	27	28	30	30
Daily UTCI	495	28	28	1.6	24	27	29	30
Agricultural employment	495	3.2	3.2	0.5	3.2	3.2	3.2	7.4
Vulnerable employment	495	19	19	2.2	19	19	19	38
Formality	0				Inf			-Inf
Wage labour	495	76	76	1.8	60	76	76	76
GNI per capita (ln)	495	9.7	9.7	0.024	9.5	9.7	9.7	9.7

Country: Tunisia

Hot week	3885	0.18	0	0.39	0	0	0	1
Threshold at 90th percentile	3885	28	28	1.4	25	28	29	34
Daily UTCI	3885	22	24	7	3.6	16	28	33
Agricultural employment	3885	15	15	0.046	15	15	15	15
Vulnerable employment	3885	20	19	0.32	19	19	19	20
Formality	0				Inf			-Inf

Wage labour	3885	74	74	0.5	73	74	74	74
GNI per capita (ln)	3885	8.3	8.3	0.058	8.2	8.3	8.3	8.4
Country: Turkey								
Hot week	4962	0.075	0	0.26	0	0	0	1
Threshold at 90th percentile	4962	26	24	3	18	24	28	33
Daily UTCI	4962	17	18	6.9	-8.7	13	21	34
Agricultural employment	4962	20	20	0.74	19	20	21	21
Vulnerable employment	4962	29	28	0.63	28	28	29	29
Formality	0				Inf			-Inf
Wage labour	4962	67	67	0.63	66	66	67	68
GNI per capita (ln)	4962	9.4	9.4	0.058	9.3	9.3	9.4	9.4
Country: Turkmenistan								
Hot week	3993	0.38	0	0.49	0	0	1	1
Threshold at 90th percentile	3993	32	32	1.2	29	31	33	35
Daily UTCI	3993	29	28	4	18	26	32	38
Agricultural employment	3993	24	24	1	23	23	25	25
Vulnerable employment	3993	33	32	1.3	31	31	35	35
Formality	0				Inf			-Inf
Wage labour	3993	65	65	1.2	63	63	66	66

GNI per capita (ln)	3993	8.8	8.8	0.045	8.8	8.8	8.9	8.9
				Country: Ukraine				
Hot week	3932	0.3	0	0.46	0	0	1	1
Threshold at 90th percentile	3932	23	23	1.4	20	22	24	26
Daily UTCI	3932	17	18	6.6	-14	13	22	30
Agricultural employment	3932	15	15	0.31	15	15	16	16
Vulnerable employment	3932	15	15	0.13	14	14	15	15
Formality	0				Inf			-Inf
Wage labour	3932	84	84	0.12	84	84	84	84
GNI per capita (ln)	3932	7.9	7.9	0.17	7.8	7.8	8.2	8.2
				Country: United Arab Emirates				
Hot week	7545	0.043	0	0.2	0	0	0	1
Threshold at 90th percentile	7545	38	38	0.76	36	38	39	40
Daily UTCI	7545	31	33	6.4	8.6	28	35	41
Agricultural employment	7545	1.9	2	0.22	1.6	1.8	2	2.2
Vulnerable employment	7545	0.48	0.4	0.14	0.34	0.4	0.47	0.71
Formality	0				Inf			-Inf
Wage labour	7545	97	97	0.2	97	97	97	97
GNI per capita (ln)	7545	11	11	0.046	11	11	11	11

Country: United Kingdom

Hot week	3711	0.043	0	0.2	0	0	0	1
Threshold at 90th percentile	3711	14	14	1.9	10	13	16	17
Daily UTCI	3711	3.8	4.3	7.6	-18	-1.9	9.6	21
Agricultural employment	3711	1.2	1.2	0.058	1.1	1.1	1.3	1.3
Vulnerable employment	3711	13	13	0.18	13	13	13	13
Formality	0				Inf			-Inf
Wage labour	3711	85	85	0.15	85	85	85	85
GNI per capita (ln)	3711	11	11	0.026	11	11	11	11

Country: United States

Hot week	5023	0.11	0	0.31	0	0	0	1
Threshold at 90th percentile	5023	26	26	4.5	11	23	30	32
Daily UTCI	5023	17	20	11	-30	11	25	34
Agricultural employment	5023	1.4	1.4	0.041	1.4	1.4	1.4	1.4
Vulnerable employment	5023	4	4.1	0.04	4	4.1	4.1	4.1
Formality	0				Inf			-Inf
Wage labour	5023	94	94	0.076	94	94	94	94
GNI per capita (ln)	5023	11	11	0.022	11	11	11	11

Country: Uruguay

Hot week	3922	0	0	0	0	0	0	0
Threshold at 90th percentile	3922	25	24	1.1	22	24	25	28
Daily UTCI	3922	8.3	7.8	4.5	-6.7	5.3	11	25
Agricultural employment	3922	8.7	8.7	0.33	8.2	8.2	9.1	9.1
Vulnerable employment	3922	23	24	0.62	22	22	24	24
Formality	3922	95	95	0	95	95	95	95
Wage labour	3922	72	72	0.49	72	72	73	73
GNI per capita (ln)	3922	9.7	9.7	0.012	9.6	9.6	9.7	9.7

Country: Uzbekistan

Hot week	3957	0.45	0	0.5	0	0	1	1
Threshold at 90th percentile	3957	29	29	2.2	23	28	30	32
Daily UTCI	3957	27	27	4	16	24	30	39
Agricultural employment	3957	27	27	0.17	27	27	28	28
Vulnerable employment	3957	38	37	1.2	36	36	38	39
Formality	0				Inf			-Inf
Wage labour	3957	62	63	1.2	61	62	64	64
GNI per capita (ln)	3957	7.9	7.9	0.048	7.8	7.8	7.9	7.9

Country: Venezuela

Hot week	3780	0.12	0	0.32	0	0	0	1
Threshold at 90th percentile	3780	29	29	4.1	15	27	31	34
Daily UTCI	3780	27	27	4.1	12	26	30	35
Agricultural employment	3780	7.4	7.4	0.051	7.3	7.3	7.4	7.4
Vulnerable employment	3780	35	36	1.8	33	34	38	38
Formality	0				Inf			-Inf
Wage labour	3780	62	62	1.4	60	60	63	64
GNI per capita (ln)	3780	9.5	9.5	0	9.5	9.5	9.5	9.5
				Country: Vietnam				
Hot week	3943	0.088	0	0.28	0	0	0	1
Threshold at 90th percentile	3943	32	33	1.6	26	32	34	35
Daily UTCI	3943	28	30	6.3	5.4	26	32	36
Agricultural employment	3943	43	42	2.3	40	42	44	46
Vulnerable employment	3943	58	56	2.8	55	56	58	62
Formality	3943	92	92	0	92	92	92	92
Wage labour	3943	40	41	2.7	36	39	41	43
GNI per capita (ln)	3943	7.6	7.6	0.044	7.5	7.6	7.6	7.7
				Country: Yemen				
Hot week	3820	0.056	0	0.23	0	0	0	1

Threshold at 90th percentile	3820	28	28	6.1	18	22	34	37
Daily UTCI	3820	26	26	6.7	8	21	31	38
Agricultural employment	3820	29	29	0.2	29	29	29	29
Vulnerable employment	3820	45	46	1.7	42	44	47	47
Formality	0				Inf			-Inf
Wage labour	3820	48	47	1.7	46	46	49	51
GNI per capita (ln)	3820	7.1	7.1	0.11	7	7	7.1	7.3
			Country: Zimbabwe					
Hot week	3834	0	0	0	0	0	0	0
Threshold at 90th percentile	3834	24	25	1.8	22	23	26	28
Daily UTCI	3834	14	15	3.7	2.3	12	16	25
Agricultural employment	3834	67	67	0.28	66	66	67	67
Vulnerable employment	3834	66	66	0.35	66	66	67	67
Formality	3834	97	97	0	97	97	97	97
Wage labour	3834	33	33	0.35	33	33	34	34
GNI per capita (ln)	3834	7.2	7.2	0.012	7.2	7.2	7.2	7.2

1.3 Distribution of food insecurity and heat variable

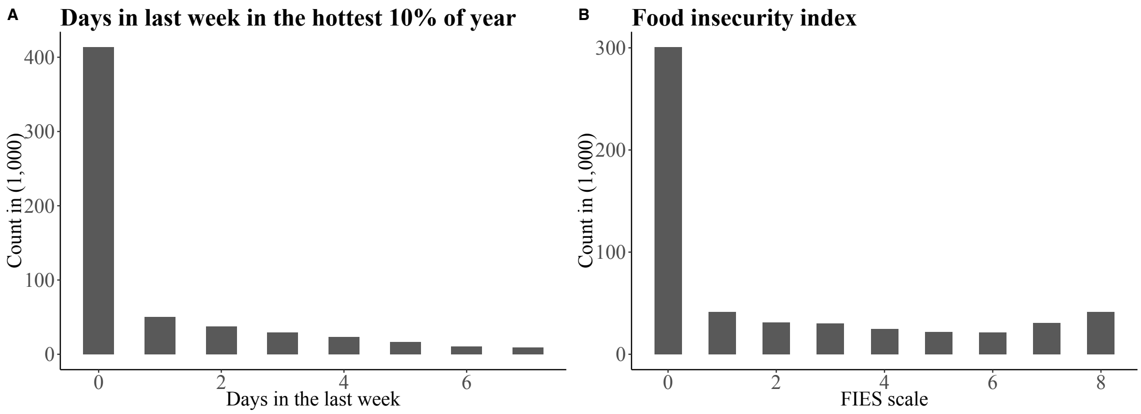

Figure 1: (A) Observations for the number of hot days in the week leading up to the survey date. (B) Observations for the number of 'Yes' responses to the food insecurity experience scale

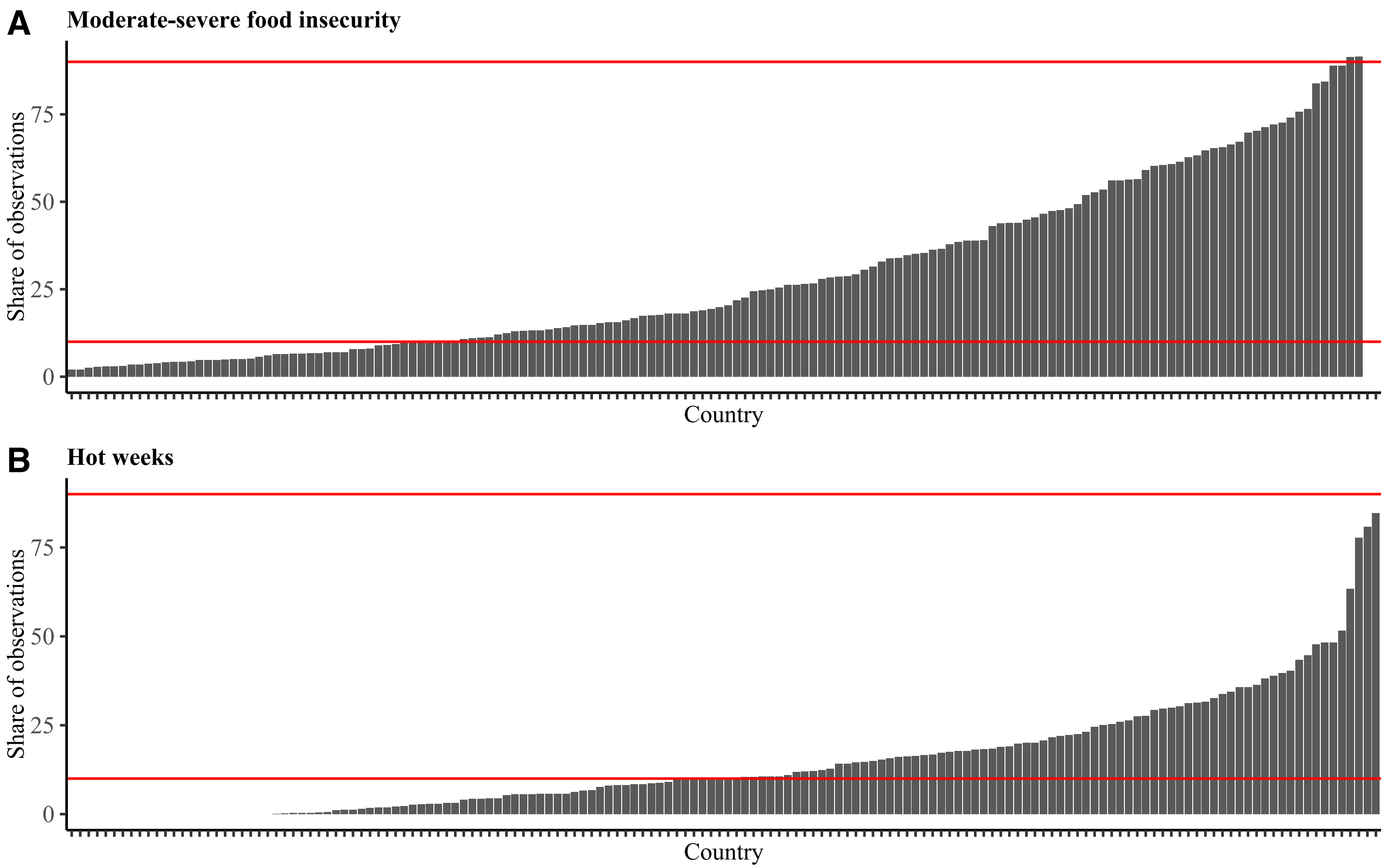

Figure 2: A) Share of moderate-severe food insecure observations by country with the red lines representing 10 and 90%. B) Share of hot week observations by country with the red lines representing 10 and 90%.

1.4 Distribution of UTCI across day, area, and year

Figure 3 shows that the majority of observations are from sub-regions with a substantial degree of seasonality throughout the year (Panel A), that almost all observations are from sub-regions within which the individual 0.25 x 0.25 longitude and latitude observations of UTCI do not vary by more than a 5°C standard deviation on a given day with a median standard deviation of 1.35°C (Panel B), and that the variation of hourly temperatures on a day in a sub-region approximates a normal distribution with a median of 6.17°C (Panel C).

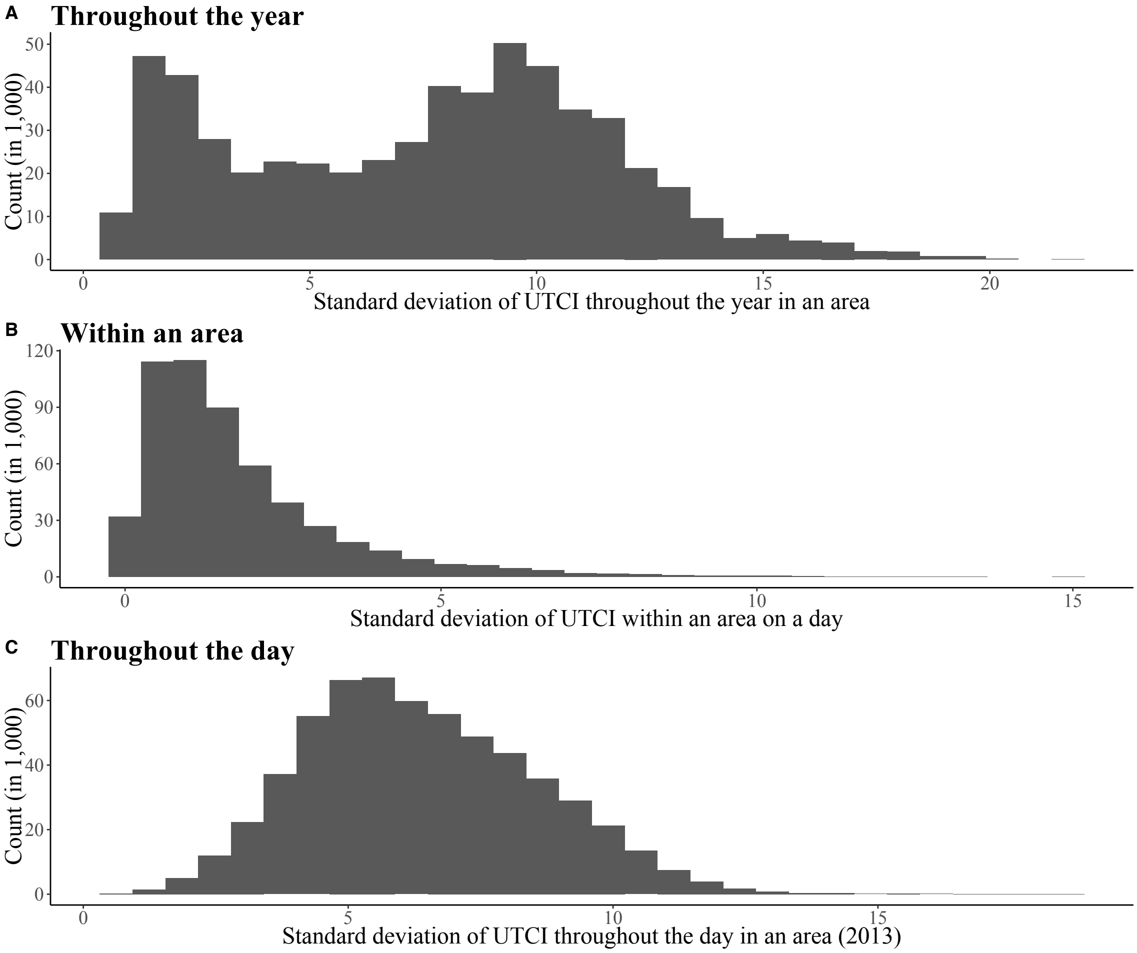

Figure 3: Standard deviation of UTCI (A) throughout the year in each sub-region, (B) within each sub-region in a day, (C) on a day within a sub-region

2 Main results

2.1 Mediation results

The table below shows that a hot week is associated with significantly (1) higher levels of un- or underemployment (2) a worse job market rating, (3) lower annual household income (ln) (4) more health problems, and (5) difficulties getting by on income. Two-sided t-tests were conducted for a 95% confidence level.

Table 5: Models testing an association between a hot week and different mediators

	Dependent variable:				
	Unemployment	Job	Income	Health	Difficulties
	(1)	(2)	(3)	(4)	(5)
Intercept	0.227*** (0.008)	0.641*** (0.019)	9.108*** (0.116)	−0.096*** (0.011)	0.273*** (0.018)
Hot week	0.004*** (0.002)	0.010*** (0.002)	−0.031*** (0.004)	0.004** (0.002)	0.013*** (0.002)
Area: Urban	−0.003*** (0.001)	−0.049*** (0.002)	0.237*** (0.003)	−0.025*** (0.002)	−0.055*** (0.002)
Age	−0.002*** (0.00003)	0.002*** (0.00005)	−0.008*** (0.0001)	0.009*** (0.00004)	0.002*** (0.00004)
Gender: Male	−0.018*** (0.001)	−0.017*** (0.002)	0.239*** (0.003)	−0.053*** (0.001)	−0.039*** (0.001)
Partner: Yes	0.006*** (0.001)	−0.023*** (0.001)	0.120*** (0.003)	−0.041*** (0.001)	−0.029*** (0.001)
Children: Yes	0.005*** (0.001)	0.004** (0.002)	0.031*** (0.003)	−0.006*** (0.001)	0.046*** (0.002)
Precipitation (ln)	0.00002 (0.0001)	−0.0001 (0.0001)	−0.001*** (0.0002)	0.0001 (0.0001)	−0.0002 (0.0001)
Hot days in last 365 days	−0.0003** (0.0001)	−0.002*** (0.0002)	−0.00005 (0.0003)	−0.0001 (0.0001)	−0.0003* (0.0002)
Year-round UTCI	0.002*** (0.0003)	0.001* (0.001)	−0.002 (0.001)	0.003*** (0.0004)	0.004*** (0.001)
2015	−0.003* (0.002)	0.007*** (0.002)	0.041*** (0.004)	0.002 (0.002)	0.009*** (0.002)
2016	0.009*** (0.002)	0.008*** (0.002)	0.017*** (0.004)	0.006*** (0.002)	0.003 (0.002)
2017	0.007*** (0.002)	−0.040*** (0.002)	0.081*** (0.004)	0.015*** (0.002)	0.013*** (0.002)
Observations	424,577	373,495	427,832	422,043	420,767
Log Likelihood	−139,151.900	−232,103.000	−534,231.700	−216,153.100	−254,400.900
Akaike Inf. Crit.	278,335.800	464,238.000	1,068,495.000	432,338.100	508,833.900
Bayesian Inf. Crit.	278,511.200	464,411.300	1,068,671.000	432,513.400	509,009.100

Note: *p<0.1; **p<0.05; ***p<0.01

Tables 6-10 show the results of the sequential causal mediation analyses as implemented in the R package mediation. The mediation package in R when applied to linear regression models uses the framework of linear structural equation modeling following Baron and Kenny [1] and developed further by Imai and colleagues [7] [8]. The procedure fits different models, specifically one model testing the relationship between the predictor and the mediator, and another model testing the predictor and the outcome variable while controlling for the mediator. The model parameters are then simulated using their approximate asymptotic distribution and the relevant causal mediation effects are computed for each draw of parameter values. Specifically, the procedure offers the total effect of the predictor on the hot week, and disaggregates this total effect into the direct and the average causal mediated effect.

In this paper, the mediators include annual household income, whether a respondents reports experiencing health problems, whether the local job market is described as good or bad, whether respondents wanted more employment than they had in the last seven days, and whether respondents report feeling difficulties getting by on their income. Analyses were conducted with complete observations for each mediator with the number of observations ranging from 424,577 for employment and 427,832 for annual income. The number of simulations for each mediation analysis is 100. Two-sided t-tests were conducted for a 95% confidence level. For example, Table 6 for annual income shows that the total effect of a hot week on food insecurity is 0.005, of which 0.0013 is a direct effect of the hot week on food insecurity, and 0.0036 is mediated through reductions in annual income. The proportion mediated indicates that 72.46% of the total effect is mediated through income and is the share of the average causal mediated effect divided by the total effect: $0.0036 \div 0.005 = 0.7246$.

Table 6: Annual income

	variable	estimate	pvalue	lowerCI	upperCI
1	Average causal mediated effect	0.0036	<0.01	0.0029	0.0044
2	Direct effect	0.0013	0.46	-0.0019	0.0039
3	Total effect	0.005	<0.01	0.0012	0.0079
4	Proportion mediated	0.7246			
5	Observations	427832			
6	Simulations	100			

Table 7: Health problems

	variable	estimate	pvalue	lowerCI	upperCI
1	Average causal mediated effect	6e-04	<0.01	1e-04	0.0011
2	Direct effect	0.004	0.06	1e-04	0.0066
3	Total effect	0.0046	0.04	6e-04	0.0076
4	Proportion mediated	0.1214			
5	Observations	422043			
6	Simulations	100			

Table 8: Difficulties getting by on income

	variable	estimate	pvalue	lowerCI	upperCI
1	Average causal mediated effect	0.0033	<0.01	0.0022	0.0043
2	Direct effect	0.0018	0.18	-0.001	0.0047
3	Total effect	0.0052	0.02	0.0021	0.008
4	Proportion mediated	0.6335			
5	Observations	420767			
6	Simulations	100			

Table 9: Local job market

	variable	estimate	pvalue	lowerCI	upperCI
1	Average causal mediated effect	8e-04	<0.01	5e-04	0.0011
2	Direct effect	0.0038	0.02	1e-04	0.008
3	Total effect	0.0046	0.02	0.0011	0.0087
4	Proportion mediated	0.1626			
5	Observations	373495			
6	Simulations	100			

Table 10: Employment

	variable	estimate	pvalue	lowerCI	upperCI
1	Average causal mediated effect	2e-04	0.12	-1e-04	6e-04
2	Direct effect	0.0048	<0.01	6e-04	0.0077
3	Total effect	0.005	<0.01	7e-04	0.008
4	Proportion mediated	0.0475			
5	Observations	424577			
6	Simulations	100			

2.2 Moderation results

Regression Table 11 below presents results from the moderation analysis testing whether the association between a hot week and moderate-severe food insecurity is moderated by (1) gross national income per capita, (2) the agricultural employment as a share of total employment, and (3) vulnerable employment as a share of total employment. Standard errors are reported next to the coefficients and two-sided t-tests were conducted for a 95% confidence level.

Table 11: Moderation analysis

	Dependent variable:		
	Moderate-severe food insecurity		
	(1)	(2)	(3)
Intercept	0.906*** (0.053)	0.771*** (0.070)	0.507*** (0.069)
Hot week	0.062*** (0.011)	−0.008** (0.003)	−0.006* (0.003)
Hot week x GNI	−0.007*** (0.001)		
Hot week x Agricultural emp.		0.001*** (0.0001)	
Hot week x Vulnerable em.			0.0003*** (0.0001)
GNI	−0.090*** (0.006)	−0.078*** (0.007)	−0.058*** (0.007)
Agricultural employment		0.001*** (0.0005)	
Vulnerable employment			0.003*** (0.0004)
Area: Urban	−0.034*** (0.001)	−0.035*** (0.001)	−0.035*** (0.001)
Age	0.001*** (0.00004)	0.001*** (0.00004)	0.001*** (0.00004)
Gender: Male	−0.024*** (0.001)	−0.024*** (0.001)	−0.024*** (0.001)
Partner: Yes	−0.017*** (0.001)	−0.017*** (0.001)	−0.017*** (0.001)
Children: Yes	0.042*** (0.001)	0.043*** (0.001)	0.043*** (0.001)
Precipitation (ln)	−0.0004*** (0.0001)	−0.0004*** (0.0001)	−0.0004*** (0.0001)
Hot days in the last 365 days	0.0002 (0.0001)	0.0002 (0.0001)	0.0002* (0.0001)
Year-round UTCI in subregion	0.006*** (0.001)	0.006*** (0.001)	0.005*** (0.001)
2015	0.007*** (0.002)	0.009*** (0.002)	0.010*** (0.002)
2016	0.026*** (0.002)	0.028*** (0.002)	0.030*** (0.002)
2017	0.048*** (0.002)	0.051*** (0.002)	0.053*** (0.002)
Number of subregions	2539	2513	2513
Number of countries	147	147	147
Standard deviation of subregions	0.0823	0.0824	0.0827
Standard deviation of countries	0.1471	0.1443	0.1417
Observations	425,375	422,377	422,377
Log Likelihood	−179,211.200	−178,346.700	−178,318.200
Akaike Inf. Crit.	358,458.500	356,731.400	356,674.400
Bayesian Inf. Crit.	358,655.800	356,939.600	356,882.600
Note:			*p<0.1; **p<0.05; ***p<0.01

3 Experiment

3.1 Covariate balance

A common concern in quasi-experimental designs and observational studies is covariate imbalance, which means that the treatment and control group are not identical with respect to important covariates that may influence the outcome and bias the estimated treatment effect [3]. For example, older people may be more likely to stay at home during hot week because they are more physiologically vulnerable and therefore may be overrepresented in the treatment group (hot week) compared to the control group (not a hot week).

A potential solution to this issue is to include covariates such as age in regression models. However, there are a few theoretical disadvantages of covariate adjustment and we therefore additionally use Covariate Balancing Propensity Scores (CBPS) [4] [3]. In essence, this procedure estimates the probability of receiving treatment based on the covariates of the treatment and control group. An algorithm then uses these probability scores to weigh the observations such that, on average, individuals with the same probability score have a similar distributions of each covariate independent of their treatment assignment [11]. The covariates are now more balanced between the treatment and control group which helps estimate a more accurate treatment effect.

Regression Table 12 displays models estimating the relationship between a hot week and (1) mild-to-severe, (2) moderate-severe, and (3) severe food insecurity using the covariate balance propensity scores. Standard errors are reported next to the coefficients and two-sided t-tests were conducted for a 95% confidence level. The effect of a hot week on food insecurity remains positive and significant.

Table 12: Regression models using covariate balance propensity scores

	Dependent variable:		
	Mild to severe	Moderate-severe	Severe
	(1)	(2)	(3)
Intercept	0.330*** (0.022)	0.132*** (0.020)	0.030* (0.016)
Hot week	0.004** (0.002)	0.005*** (0.001)	0.007*** (0.001)
Area: Urban	−0.033*** (0.002)	−0.033*** (0.001)	−0.022*** (0.001)
Age	0.001*** (0.00004)	0.001*** (0.00004)	0.001*** (0.00003)
Gender: Male	−0.027*** (0.001)	−0.024*** (0.001)	−0.013*** (0.001)
Partner: Yes	−0.025*** (0.001)	−0.016*** (0.001)	−0.008*** (0.001)
Children: Yes	0.049*** (0.002)	0.037*** (0.001)	0.018*** (0.001)
Precipitation (ln)	−0.0004*** (0.0001)	−0.0003*** (0.0001)	−0.0002** (0.0001)
Year-round UTCI	0.006*** (0.001)	0.006*** (0.001)	0.005*** (0.0005)
Hot days in past year	0.00003 (0.0001)	0.0001 (0.0001)	0.0003*** (0.0001)
2015	−0.008*** (0.002)	0.003* (0.002)	0.004*** (0.001)
2016	0.009*** (0.002)	0.022*** (0.002)	0.019*** (0.001)
2017	0.027*** (0.002)	0.043*** (0.002)	0.035*** (0.002)
Number of subregions	2557	2557	2557
Number of countries	149	149	149
Standard deviation of subregions	0.0955	0.0897	0.0756
Standard deviation of countries	0.2254	0.207	0.1558
Observations	427,832	427,832	427,832
Log Likelihood	−306,326.300	−242,397.700	−147,538.000
Akaike Inf. Crit.	612,684.600	484,827.400	295,108.000
Bayesian Inf. Crit.	612,860.000	485,002.900	295,283.500

Note: *p<0.1; **p<0.05; ***p<0.01

3.2 Selection bias

There may be a selection bias into the treatment and control group. As mentioned in the previous section, older people may be more likely to be interviewed on hot days because they are physiologically more sensitive to heat and may not be able to work or have to stay in cooler indoor surroundings. The CBPS scores used in the previous section and the main manuscript already account for potential imbalances around age, but an important factor that they do not consider is income. There may be a higher share of interviews with low incomes on hotter days because people on lower incomes may be more likely to be in heat exposed forms of employment, such as agriculture. Since income is a potential mediator, it is not included in the main model as a covariate and therefore not included in the CBPS. As an alternative approach, I analyse whether respondents select into the treatment and control group based on their income by calculating the total number of interviews on an interview day and the share of interviews with lower incomes.

Table 13 shows the results. In model (1) I use the number of interviews as the dependent variable and in model (2) I use the share of low income interviewees and test whether a hot week has a significant effect on each. Standard errors are reported next to the coefficients and two-sided t-tests were conducted for a 95% confidence level. I find that a hot week is associated with a significantly lower number of interviews and a significantly higher share of interviews with low incomes. Low incomes are defined as the lowest quartile of annual household income in the sub-region in that year. In model (3) I then include the share of low income interviews a a control variate in the main model and find that the association between a hot week and food insecurity still holds. I do not include the number of interviews because it is not likely to confound the main relationship.

Table 13: Models exploring the selection bias

	Dependent variable:		
	Number of interviews	Share of low income interviews	Moderate-severe food insecurity
	(1)	(2)	(3)
Intercept	9.473*** (0.576)	0.265*** (0.005)	0.079*** (0.020)
Hot week	−0.834*** (0.037)	0.003*** (0.001)	0.007*** (0.002)
Low income interviews share			0.174*** (0.003)
Area: Urban	−0.250*** (0.029)	−0.049*** (0.001)	−0.025*** (0.001)
Age	0.0001 (0.001)	0.001*** (0.00002)	0.001*** (0.00004)
Gender: Male	0.023 (0.025)	−0.034*** (0.001)	−0.018*** (0.001)
Partner: Yes	−0.001 (0.023)	−0.012*** (0.001)	−0.015*** (0.001)
Children: Yes	−0.081*** (0.027)	−0.001 (0.001)	0.043*** (0.001)
Precipitation (ln)	−0.004** (0.002)	−0.0001** (0.0001)	−0.0004*** (0.0001)
Hot days in last 365 days	0.005* (0.003)	−0.0003*** (0.0001)	0.0002* (0.0001)
Year-round UTCI	0.054*** (0.020)	0.0003 (0.0002)	0.006*** (0.001)
2015	0.055 (0.034)	0.001 (0.001)	0.009*** (0.002)
2016	−0.738*** (0.033)	0.002*** (0.001)	0.031*** (0.002)
2017	−0.702*** (0.034)	0.001 (0.001)	0.053*** (0.002)
Observations	460,162	460,162	427,832
Log Likelihood	−1,595,072.000	81,488.890	−177,757.100
Akaike Inf. Crit.	3,190,177.000	−162,945.800	355,548.300
Bayesian Inf. Crit.	3,190,353.000	−162,769.100	355,734.700

Note: *p<0.1; **p<0.05; ***p<0.01

3.3 Geographic match

A limitation in this analysis is that the granularity of the geographic level is determined by the size of the sub-regional unit in the Gallup World Poll. This means that the UTCI for observations is an average over all the observations within a sub-region. The sub-regional unit is often at the administrative level of the state. For the United Kingdom, this is England, Wales, Scotland, and Northern Ireland.

Panel A is an example plotting the UTCI longitude and latitude observations from the ERA-5 HEAT data set for the United Kingdom to give an impression of the granularity of the UTCI observations and the varying sizes of the state-level administrative units. Panel B) is a boxplot of the size of the administrative units. The average size of a state-level administrative unit across all observations was 9.40 km^2 (ln) with a first quartile of 8.09 km^2, median of 9.43 km^2, and third quartile of 10.66 km^2 and a maximum of 14.37 km^2. This means that the median observation is a little larger than half the size of Wales, England.

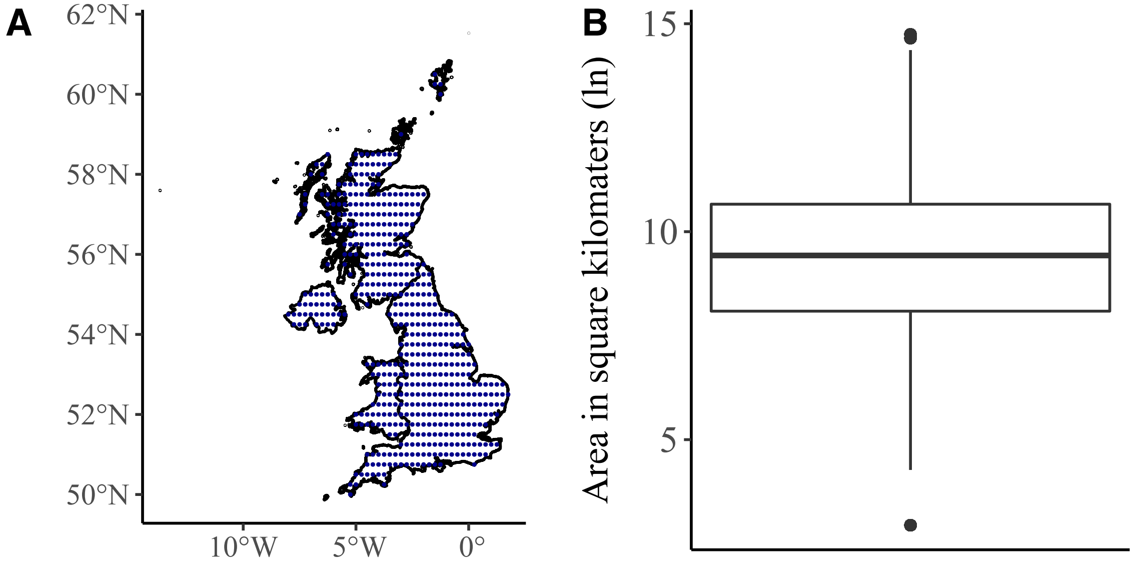

Figure 4: A) UTCI observations in the United Kingdom, B) Boxplot for the average size (km^2, ln) of an administrative unit across all observations, lower whisker = 2.94, min = 4.27 km^2, first quartile = 8.09 km^2, median = 9.43 km^2, third quartile = 10.66 km^2, max = 14.37 km^2, upper whisker = 14.74, n = 589,682

To test whether the variation of temperature within sub-regions and in particular within larger sub-regions influences the results, I have fit two separate models that are displayed in Table 14. Model (1)

shows the results for a model using only observations from sub-regions in which the UTCI observations from the longitude and latitude within the state varied by less than 2ºC standard deviation. Model (2) controls both for variation of UTCI within an area and the size of a sub-region. Standard errors are reported next to the coefficients and two-sided t-tests were conducted for a 95% confidence level.

The effect of a hot week on food insecurity from model (1) are positive and significant when using only observations with lower standard deviations of temperature within the state. In fact, the effect size is increased substantially compared to the model using all observations. The results in model (2) also indicate that the effect of a hot week remains positive, significant, and increases in size compared to the main model.

Table 14: Models using more precise geographic UTCI matches

	Dependent variable:	
	Moderate-severe food insecurity	
	(1)	(2)
Intercept	0.075*** (0.022)	0.092*** (0.030)
Hot week	0.011*** (0.002)	0.009*** (0.002)
SD area		0.002* (0.001)
Size area (ln)		0.003 (0.002)
Area: Urban	−0.032*** (0.002)	−0.035*** (0.001)
Age	0.001*** (0.00004)	0.001*** (0.00004)
Gender: Male	−0.025*** (0.002)	−0.023*** (0.001)
Partner: Yes	−0.016*** (0.001)	−0.017*** (0.001)
Children: Yes	0.039*** (0.002)	0.043*** (0.001)
Precipitation (ln)	−0.0002 (0.0001)	−0.0004*** (0.0001)
Hot days in the last 365 days	0.001*** (0.0002)	0.0002 (0.0001)
Year-round UTCI in subregion	0.008*** (0.001)	0.006*** (0.001)
2015	0.010*** (0.002)	0.010*** (0.002)
2016	0.038*** (0.002)	0.032*** (0.002)
2017	0.054*** (0.002)	0.053*** (0.002)
Observations	281,069	420,679
Log Likelihood	−115,594.400	−176,647.700
Akaike Inf. Crit.	231,220.800	353,331.400
Bayesian Inf. Crit.	231,389.600	353,528.500
Note:		*p<0.1; **p<0.05; ***p<0.01

It is also important to note that there are a few irregularities in the administrative unit of the Gallup

World Poll which seem to have been driven by context-specific considerations. For example, the sample for the United Kingdom also includes London which is not mutually exclusive to England. While this presented a lot of challenges during the initial data wrangling, I decided to use this additional layer of information because it provided an opportunity to find more precise UTCI estimates for observations. To find the most precise longitude and latitude match for each context-specific sub-region, I used an API request for Google Maps that returned the longitude and latitude of each sub-regional identifier (e.g., 'London, England'). I then matched 'London, England' not to the average UTCI for England, but to the closest longitude and latitude observation from the ERA-5 HEAT data set. I then proceeded with the analysis using averages for sub-regions and exact matches when a context-specific sub-region was available.

4 Model specifications

4.1 Non-linearity

Current approaches in environmental epidemiology often use non-linear generalized models and in particular distributed lag non-linear models (DLNM). A DLNM is usually fit on long-term daily time series data. However, this data is in a panel format where different sub-regions are interviewed at different times of the year over short periods. Therefore, DLNMS do not apply to the format of this data set. Instead, I fit separate models with non-linear specifications. I also explore lags separately later in this appendix.

Table 15 presents results from (1) a Logit model to estimate the relationship between moderate-severe food insecurity and a hot week, (2) a Poisson model between the number of 'yes' responses to the eight questions on the FIES and a hot week, and (3) a Poisson model between the number of 'yes' responses to the eight questions on the FIES and the percentile of UTCI on the survey date, and (4) a Poisson model between the number of 'yes' responses to the eight questions on the FIES and the average UTCI on the survey date. Standard errors are reported next to the coefficients and two-sided t-tests were conducted for a 95% confidence level. The positive and significant association between heat and food insecurity holds across logit and poisson models using different measures of heat and food insecurity.

The model presented in the main manuscript is a multi-level linear probability for binary response. The model performs en par with a logit non-linear model. The Akaike Information Criterion for the linear probability model is 359,406 compared to an AIC of 364,961 for the logit model presented in Table 15 model 1. A linear model therefore aligns with the results from non-linear models, performs en par with non-linear models, is computationally almost 55 times more efficient than non-linear models, and is easy to interpret and understand for a range of audiences.

Table 15: Non-linear models

	Dependent variable:			
	Moderate-severe FI	Index		
	(1)	(2)	(3)	(4)
Intercept	−3.4872*** (0.0598)	−0.8318*** (0.0294)	−0.8480*** (0.0296)	2.3461*** (0.0883)
Hot week	0.0529*** (0.0133)	0.0214*** (0.0035)		
Percentile on day			0.0002*** (0.0001)	
UTCI on day				0.0011*** (0.0003)
Area: Urban	−0.2846*** (0.0113)	−0.1446*** (0.0031)	−0.1446*** (0.0031)	−0.1416*** (0.0031)
Age	0.0078*** (0.0003)	0.0038*** (0.0001)	0.0038*** (0.0001)	0.0038*** (0.0001)
Gender: Male	−0.1811*** (0.0092)	−0.0867*** (0.0024)	−0.0867*** (0.0024)	−0.0861*** (0.0024)
Partner: Yes	−0.1253*** (0.0085)	−0.0631*** (0.0022)	−0.0631*** (0.0022)	−0.0639*** (0.0022)
Children: Yes	0.3143*** (0.0100)	0.1487*** (0.0027)	0.1487*** (0.0027)	0.1477*** (0.0027)
GNI per capita (ln)				−0.3211*** (0.0086)
Precipitation (ln)	−0.0032*** (0.0007)	−0.0012*** (0.0002)	−0.0012*** (0.0002)	−0.0012*** (0.0002)
Year-round UTCI	0.0021** (0.0009)	0.0007*** (0.0002)	0.0008*** (0.0002)	0.0008*** (0.0002)
Hot days in last 365 days	0.0565*** (0.0130)	0.0098*** (0.0035)	0.0108*** (0.0035)	0.0083** (0.0035)
2015	0.2128*** (0.0129)	0.0882*** (0.0035)	0.0889*** (0.0035)	0.0810*** (0.0035)
2016	0.3857*** (0.0127)	0.1642*** (0.0033)	0.1643*** (0.0034)	0.1501*** (0.0034)
2017	0.1013*** (0.0024)	0.0598*** (0.0014)	0.0598*** (0.0014)	0.0341*** (0.0014)
Model type	Logit	Poisson	Poisson	Poisson
Observations	427,832	427,832	427,832	425,375
Log Likelihood	−182,465.9000	−852,640.4000	−852,649.4000	−848,878.9000
Akaike Inf. Crit.	364,961.7000	1,705,311.0000	1,705,329.0000	1,697,790.0000
Bayesian Inf. Crit.	365,126.2000	1,705,475.0000	1,705,493.0000	1,697,965.0000
Note:				*p<0.1; **p<0.05; ***p<0.01

4.2 Continuous heat measures

The model presented in the main manuscript uses a binary indicator to capture whether the last week was a hot week. As a reminder, the definition for a hot week is whether at least three days in the last week were in the hottest 90% of the year in that sub-region.

To ensure that the results hold across a range of heat measures, I explore continuous heat measures in this section. Table 16 below shows the results from multi-level linear regression models for the association between moderate-severe food insecurity and (1) UTCI on the day of the interview, and (2) percentile of UTCI on the day of the interview in that year and sub-region. Standard errors are reported next to the coefficients and two-sided t-tests were conducted for a 95% confidence level.

Model (1) shows that UTCI on the day of the interview has a slightly negative and insignificant effect of -0.0001 [95%-CI: -0.00032 - 0.00015, t=-0.687, p=0.492] on food insecurity. Model (2) shows that percentile on the day of the interview has a slightly positive and significant effect of 0.0001 [95%-CI: 1.08e-06 - 0.0001, t=1.998, p=0.0457] on food insecurity. Model (3) bins UTCI on the day of the survey into 4°C buckets and shows that moderate-severe food insecurity is significantly higher on days in buckets from 18-38°C compared to days with average UTCIs from 14-18^{a}C.

Model (1) indicates an insignificant negative association between food insecurity and heat, while model (2) indicates a significant positive association. Model (1) may show results inconsistent with the main theory that heat increases food insecurity because continuous heat measurements are unlikely to have a linear association with food insecurity. This becomes clear when looking at model (3) again where both higher and lower values of UTCI have a significantly positive effect on food insecurity. Therefore, using a linear approach to modelling a continuous heat measure and food insecurity may not be appropriate, and a Logit or Poisson regression may be more appropriate. Indeed, Table 13 model 4 presented a non-linear Poisson models that tested the association between the FIES scale and UTCI on the day, finding that higher levels of heat on the day of the interview are associated with significantly higher levels of food insecurity.

Table 16: Models using continuous heat measures

	Dependent variable:		
	Moderate-severe food insecurity		
	(1)	(2)	(3)
Intercept	0.920*** (0.053)	0.117*** (0.020)	0.918*** (0.053)
Daily UTCI	−0.0001 (0.0001)		
Daily Percentile		0.0001** (0.00003)	
(-26,-22]			0.003 (0.050)
(-22,-18]			0.023 (0.027)
(-18,-14]			0.037** (0.018)
(-14,-10]			0.009 (0.013)
(-10,-6]			0.012 (0.008)
(-6, -2]			0.013** (0.006)
(-2, 2]			0.013*** (0.004)
(2, 6]			0.014*** (0.003)
(6, 10]			0.010*** (0.003)
(10, 14]			−0.001 (0.002)
(18, 22]			0.006** (0.002)
(22, 26]			0.009*** (0.003)
(26, 30]			0.011*** (0.003)
(30, 34]			0.008** (0.004)
(34, 38]			0.013** (0.006)
(38, 42]			0.030 (0.039)
Area: Urban	−0.034*** (0.001)	−0.035*** (0.001)	−0.034*** (0.001)
Age	0.001*** (0.00004)	0.001*** (0.00004)	0.001*** (0.00004)
Gender: Male	−0.024*** (0.001)	−0.024*** (0.001)	−0.023*** (0.001)
Partner: Yes	−0.017*** (0.001)	−0.017*** (0.001)	−0.017*** (0.001)
Children: Yes	0.042*** (0.001)	0.042*** (0.001)	0.042*** (0.001)
GNI per capita (ln)	−0.092*** (0.006)		−0.092*** (0.006)
Precipitation (ln)	−0.0004*** (0.0001)	−0.0004*** (0.0001)	−0.0004*** (0.0001)
Year-round UTCI	0.0002* (0.0001)	0.0002* (0.0001)	0.0003** (0.0001)
Hot days in the last 365 days	0.006*** (0.001)	0.006*** (0.001)	0.006*** (0.001)
Observations	425,375	427,832	425,370
Log Likelihood	−179,228.200	−179,697.900	−179,263.100
Akaike Inf. Crit.	358,490.300	359,427.800	358,590.100
Bayesian Inf. Crit.	358,676.700	359,603.300	358,940.900
Note:			*p<0.1; **p<0.05; ***p<0.01
			Fixed effects for the year are not displayed in table

4.3 Absolute and relative heat measures

In this section, I explore how the results change with the choice of absolute or relative heat measures and thresholds.

Thermal comfort is a highly subjective experience that depends on individual physiology, acclimatization, and culture and is therefore difficult to model quantitatively [10]. A key distinction exists between absolute (ºC) and relative (percentile) measures of heat. I argue that a relative threshold is preferable for three main reasons.

First, it allows for acclimatization to heat which means that people who have lived in hotter climates for longer are able to physiologically tolerate higher heat levels because their bodies physiologically adapt to the heat. These people would therefore be more likely to tolerate hotter temperatures without substantial health consequences that affect their income earning abilities. For example, hospitalizations increase significantly at 27ºC in colder parts of the US but at 40ºC in hotter parts of the US [13]. This relative modeling of heat stress is also in line with the World Meteorological Organization's definition of heat waves as periods of *unusually* hot weather [5]. A relative approach is also used in other cross-national studies of heat stress [9, 6].

Second, the relative thresholds used in this data correspond to absolute heat levels that can reasonably be considered strenuous for heat-exposed workers. The absolute UTCI values that underly the relative thresholds in each sub-region are displayed in Figure 5 for the 95th percentile in Panel A and the 95th percentile in Panel B. For 50% of observations, the 90th percentile corresponds to an absolute UTCI equal or higher to 26.6ºC. For 25% of observations, the absolute UTCI threshold underlying the 90th percentile is equal or higher to 30.8ºC. Moreover, these values are average daily UTCIs and the daily maximum is likely to be even higher.

Finally, a relative measure is likely to yield a more conservative measure of the effect of a hot week on food insecurity. This is because some weeks will be considered hot that were rather cool. For example, hot weeks in Iceland were on average based on days crossing 12ºC. Considering weeks as hot that likely

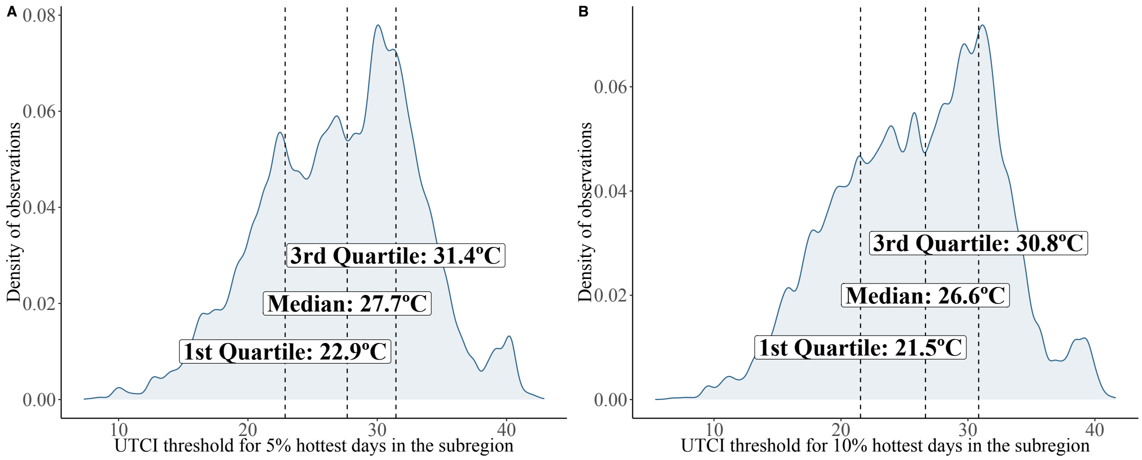

Figure 5: A) Density of observations for the absolute values underlying the 95th percentile across all subregions. B) Density of observations for the absolute values underlying the 90th percentile across all subregions.

were not perceived to be hot by the local population will bias the estimate towards zero. I am therefore choosing an approach to modelling heat that errs on the side of caution.

The models in Table 17 use different relative heat thresholds to define a hot week. Model (1) uses the hottest 5% of UTCI in a year in a sub-region to define a hot day and a hot week. Model (2) uses the hottest 10%, and Model (3) uses the hottest 15%. Standard errors are reported next to the coefficients and two-sided t-tests were conducted for a 95% confidence level. The results suggest that the choice of a relative threshold does not influence the results of the main model. In line with expectations, the effect sizes become stronger for higher thresholds. For example, the effect of a hot week for the hottest 5% of days is 0.0055 [95%-CI: 0.0009 - 0.0101, t=2.36, p=0.0183], compared to an effect of 0.0044 [95%-CI: 0.0014 - 0.0075, t=2.868, p=0.0041] for a hot week using the hottest 15% of days which is approximately 20% smaller.

Table 17: Models using relative heat measures

	Dependent variable:		
	Moderate-severe food insecurity		
	(1)	(2)	(3)
Intercept	0.120*** (0.020)	0.121*** (0.020)	0.120*** (0.020)
Hot week: 05P	0.006** (0.002)		
Hot week: 10P		0.007*** (0.002)	
Hot week: 15P			0.004*** (0.002)
Area: Urban	−0.035*** (0.001)	−0.035*** (0.001)	−0.035*** (0.001)
Age	0.001*** (0.00004)	0.001*** (0.00004)	0.001*** (0.00004)
Gender: Male	−0.024*** (0.001)	−0.024*** (0.001)	−0.024*** (0.001)
Partner: Yes	−0.017*** (0.001)	−0.017*** (0.001)	−0.017*** (0.001)
Children: Yes	0.042*** (0.001)	0.042*** (0.001)	0.042*** (0.001)
Precipitation (ln)	−0.0004*** (0.0001)	−0.0004*** (0.0001)	−0.0004*** (0.0001)
Year-round UTCI	0.0002 (0.0001)	0.0002 (0.0001)	0.0002 (0.0001)
Hot days in the last 365 days	0.010*** (0.002)	0.010*** (0.002)	0.010*** (0.002)
2015	0.032*** (0.002)	0.032*** (0.002)	0.032*** (0.002)
2016	0.053*** (0.002)	0.053*** (0.002)	0.053*** (0.002)
2017	0.006*** (0.001)	0.006*** (0.001)	0.006*** (0.001)
Observations	427,832	427,832	427,832
Log Likelihood	−179,692.700	−179,687.000	−179,691.800
Akaike Inf. Crit.	359,417.400	359,405.900	359,415.600
Bayesian Inf. Crit.	359,592.900	359,581.400	359,591.100
Note:			*p<0.1; **p<0.05; ***p<0.01

The models in Table 18 use different absolute heat measures to define a hot week. Standard errors are reported next to the coefficients and two-sided t-tests were conducted for a 95% confidence level. These models are additionally controlling for the gross national income per capita because many countries with hotter year-round temperatures tend to have lower GNIs per capita and therefore higher levels of food insecurity. In contrast, the relative thresholds are not substantially correlated with GNI per capita and it is therefore not included as a covariate in the relative models.

Table 18: Models using absolute heat measures

	Dependent variable:		
	Moderate-severe food insecurity		
	(1)	(2)	(3)
Intercept	0.920*** (0.053)	0.920*** (0.053)	0.922*** (0.053)
Hot week: 30	0.001 (0.003)		
Hot week: 28		0.001 (0.003)	
Hot week: 26			0.003 (0.003)
Area: Urban	−0.034*** (0.001)	−0.034*** (0.001)	−0.034*** (0.001)
Age	0.001*** (0.00004)	0.001*** (0.00004)	0.001*** (0.00004)
Gender: Male	−0.024*** (0.001)	−0.024*** (0.001)	−0.023*** (0.001)
Partner: Yes	−0.017*** (0.001)	−0.017*** (0.001)	−0.017*** (0.001)
Children: Yes	0.042*** (0.001)	0.042*** (0.001)	0.042*** (0.001)
GNI per capita	−0.092*** (0.006)	−0.092*** (0.006)	−0.092*** (0.006)
Precipitation (ln)	−0.0004*** (0.0001)	−0.0004*** (0.0001)	−0.0004*** (0.0001)
Year-round UTCI	0.0002* (0.0001)	0.0002* (0.0001)	0.0002* (0.0001)
Hot days in the last 365 days	0.008*** (0.002)	0.008*** (0.002)	0.008*** (0.002)
2015	0.026*** (0.002)	0.026*** (0.002)	0.026*** (0.002)
2016	0.048*** (0.002)	0.048*** (0.002)	0.048*** (0.002)
2017	0.006*** (0.001)	0.006*** (0.001)	0.006*** (0.001)
Observations	425,375	425,375	425,375
Log Likelihood	−179,225.200	−179,225.200	−179,224.600
Akaike Inf. Crit.	358,484.400	358,484.400	358,483.200
Bayesian Inf. Crit.	358,670.800	358,670.700	358,669.500
Note:			*p<0.1; **p<0.05; ***p<0.01

Model (1) uses a threshold of 30ºC to define a hot day and therefore hot week and finds an effect of 0.0013 [95%-CI: -0.0037 - 0.0063, t = 0.513, p = 0.6081] for a hot week on moderate-severe food insecurity. Model (2) uses a threshold of 28ªC and finds an effect of 0.0015 [95%-CI: -0.0034 - 0.0065, t = 0.58, p = 0.5620], and model (3) uses a threshold of 26ºC and finds an effect of 0.0032 [95%-CI: -0.0020 - 0.0084, t =

1.203, p = 0.2291].

The results suggest positive but insignificant effects of heat stress on food insecurity for absolute values. One potential reason for this is that a high absolute threshold such as 30°C classifies very few observations as a hot week (see Figure 6) and classifies entire regions, such as Central and Western Europe, Southern Africa, and Northern America, and Southern America, to never have experienced a hot week. This also suggests that a relative measure is preferable: Using a high absolute threshold means that some regions, such as Europe, are never considered hot, even though heat stress and heat-related mortality are well documented issues in these regions [12].

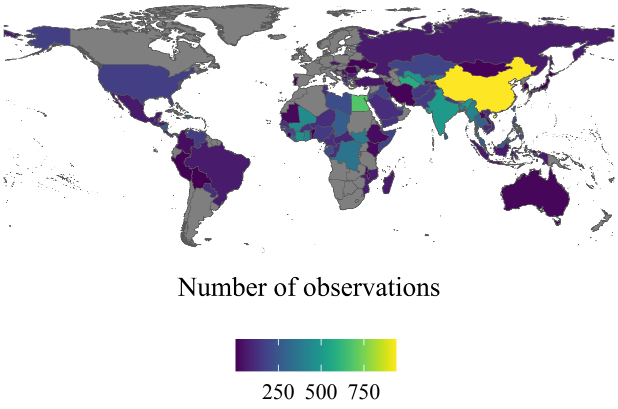

Figure 6: Plot showing the number of observations classified as a hot week by country when using a threshold of 30°C

Another explanation is that there is a high correlation between the absolute hot week indicators and the year-round UTCI, since hotter countries are more likely to qualify with an absolute threshold. For example, the Pearson correlation between a hot week using a 26°C threshold and the year-round temperature is 0.6664. Such collinearity can influence the size and precision of the estimate. Indeed, when removing the year-round UTCI as a covariate from the model, the effects of a hot week are positive and significant in all absolute models (see Table 19). In Table 19, standard errors are reported next to the coefficients and two-sided t-tests were conducted for a 95% confidence level.

Table 19: Models using absolute heat measures without controlling for year-round UTCI

	Dependent variable:		
	Moderate-severe food insecurity		
	(1)	(2)	(3)
Intercept	0.685*** (0.036)	0.684*** (0.036)	0.688*** (0.036)
Hot week: 30	0.013*** (0.002)		
Hot week: 28		0.014*** (0.002)	
Hot week: 26			0.019*** (0.002)
Area: Urban	−0.042*** (0.001)	−0.042*** (0.001)	−0.042*** (0.001)
Age	0.001*** (0.00004)	0.001*** (0.00004)	0.001*** (0.00004)
Gender: Male	−0.024*** (0.001)	−0.024*** (0.001)	−0.024*** (0.001)
Partner: Yes	−0.016*** (0.001)	−0.016*** (0.001)	−0.016*** (0.001)
Children: Yes	0.046*** (0.001)	0.046*** (0.001)	0.046*** (0.001)
GNI per capita	−0.054*** (0.004)	−0.054*** (0.004)	−0.054*** (0.004)
Precipitation (ln)	−0.0002*** (0.0001)	−0.0003*** (0.0001)	−0.0002*** (0.0001)
Hot days in the last 365 days	0.0003** (0.0001)	0.0003** (0.0001)	0.0003** (0.0001)
2015	0.009*** (0.002)	0.009*** (0.002)	0.009*** (0.002)
2016	0.030*** (0.002)	0.030*** (0.002)	0.030*** (0.002)
2017	0.050*** (0.002)	0.050*** (0.002)	0.050*** (0.002)
Observations	425,375	425,375	425,375
Log Likelihood	−183,629.300	−183,623.800	−183,605.500
Akaike Inf. Crit.	367,288.600	367,277.600	367,241.000
Bayesian Inf. Crit.	367,453.000	367,442.000	367,405.400
Note:			*p<0.1; **p<0.05; ***p<0.01

4.4 Food insecurity measures

Regression Table 20 shows models using different measures of food insecurity for the dependent variable and retaining the hot week as the variable of interest. Model (1) uses the number of 'yes' responses to the eight questions of the FIES. Model (2) uses a mild-to-severe measure of food insecurity which corresponds to at least one 'yes' responses to the FIES. Model (3) uses a moderate-to-severe measure of food insecurity which corresponds to at least four 'yes' responses to the FIES. Model (4) uses a severe measure of food insecurity which corresponds to at least seven 'yes' responses to the FIES. All models use demographic and weather-related covariates and fixed effects for the year. Standard errors are reported next to the coefficients and two-sided t-tests were conducted for a 95% confidence level. The association between a hot week and food insecurity is positive and significant for all models with effect sizes increasing for more severe forms of food insecurity. The CBPS-adjusted results for different degrees of severity of food insecurity were presented earlier in the Appendix in section 3.1

Table 20: Different measures of food insecurity

	Dependent variable:			
	Index	Mild-to-severe	Moderate-to-severe	Severe
	(1)	(2)	(3)	(4)
Intercept	1.028***	0.338***	0.121***	0.026*
	(0.137)	(0.022)	(0.020)	(0.015)
Hot week	0.058***	0.005**	0.007***	0.009***
	(0.011)	(0.002)	(0.002)	(0.001)
Area: Urban	−0.243***	−0.038***	−0.035***	−0.023***
	(0.009)	(0.002)	(0.001)	(0.001)
Age	0.008***	0.001***	0.001***	0.001***
	(0.0002)	(0.00004)	(0.00004)	(0.00003)
Gender: Male	−0.173***	−0.030***	−0.024***	−0.015***
	(0.007)	(0.001)	(0.001)	(0.001)
Partner: Yes	−0.122***	−0.025***	−0.017***	−0.008***
	(0.007)	(0.001)	(0.001)	(0.001)
Children: Yes	0.300***	0.055***	0.042***	0.024***
	(0.008)	(0.002)	(0.001)	(0.001)
Precipitation (ln)	−0.002***	−0.001***	−0.0004***	−0.0001
	(0.001)	(0.0001)	(0.0001)	(0.0001)
Year-round UTCI	0.044***	0.006***	0.006***	0.004***
	(0.004)	(0.001)	(0.001)	(0.0004)
Observations	427,832	427,832	427,832	427,832
Log Likelihood	−948,198.000	−237,759.400	−179,687.000	−87,636.970
Akaike Inf. Crit.	1,896,428.000	475,550.700	359,405.900	175,305.900
Bayesian Inf. Crit.	1,896,604.000	475,726.200	359,581.400	175,481.400

Note: *p<0.1; **p<0.05; ***p<0.01
Models include fixed effects for the year

5 Temporal effects

In this section, I fit different models that explore the role of seasonality or more long-term effects of heat, and the role of short-term effects of heat for food insecurity.

5.1 Long term effects

Table 21 shows regression models controlling for long-term effects with different categorical variables. Model (1) controls for the relative season which is defined as the coldest, second coldest, second hottest, and hottest four months of the year in that sub-region. Model (2) controls for the months of the year, e.g., February or March. The association between a hot week and moderate-to-severe food insecurity holds across both models. Standard errors are reported next to the coefficients and two-sided t-tests were conducted for a 95% confidence level.

Table 21: Models using fixed effects for months and season

	Dependent variable:	
	Moderate-severe food insecurity	
	(1)	(2)
Intercept	0.117*** (0.028)	0.108*** (0.020)
Hot week	0.006*** (0.002)	0.007*** (0.002)
Area: Urban	−0.034*** (0.001)	−0.035*** (0.001)
Age	0.001*** (0.00004)	0.001*** (0.00004)
Gender: Male	−0.017*** (0.001)	−0.017*** (0.001)
Children <15 in HH	0.042*** (0.001)	0.042*** (0.001)
Having a partner	−0.024*** (0.001)	−0.024*** (0.001)
Precipitation (ln)	−0.0004*** (0.0001)	−0.0004*** (0.0001)
Year-round UTCI in subregion	0.006*** (0.001)	0.006*** (0.001)
Hot days in the last 365 days	0.0002* (0.0001)	0.0002 (0.0001)
Second coldest season		0.006** (0.003)
Second hottest season		0.015*** (0.003)
Hottest season		0.019*** (0.004)
February	0.006 (0.022)	
March	−0.015 (0.021)	
April	−0.009 (0.020)	
May	−0.008 (0.020)	
June	0.003 (0.021)	
July	0.013 (0.021)	
August	−0.002 (0.021)	
September	0.0003 (0.021)	
October	0.008 (0.021)	
November	−0.0004 (0.021)	
December	0.024 (0.021)	
2015	0.015*** (0.002)	0.014*** (0.002)
2016	0.039*** (0.002)	0.040*** (0.002)
2017	0.059*** (0.002)	0.060*** (0.002)
Observations	427,832	427,832
Log Likelihood	−179,676.600	−179,681.900
Akaike Inf. Crit.	359,407.200	359,401.800
Bayesian Inf. Crit.	359,703.300	359,610.100
Note:		*p<0.1; **p<0.05; ***p<0.01

Table 22 shows time-stratified regression models. Model (1) stratifies by the relative season nested in the year. Model (2) stratifies by the month nested within the year. Model (2) is rank deficient and drops 5 columns. Standard errors are reported next to the coefficients and two-sided t-tests were conducted for a 95% confidence level. The association between a hot week and moderate-severe food insecurity holds across both models.

Table 22: Time-stratified models

	Dependent variable:	
	Moderate-severe food insecurity	
	(1)	(2)
Intercept	0.021 (0.025)	0.158*** (0.036)
Hot week	0.007*** (0.002)	0.006*** (0.002)
Area: Urban	−0.034*** (0.001)	−0.034*** (0.001)
Age	0.001*** (0.00004)	0.001*** (0.00004)
Gender: Male	−0.017*** (0.001)	−0.017*** (0.001)
Children <15 in HH	0.042*** (0.001)	0.042*** (0.001)
Having a partner	−0.024*** (0.001)	−0.024*** (0.001)
Precipitation (ln)	−0.0004*** (0.0001)	−0.0004*** (0.0001)
Year-round UTCI in subregion	0.006*** (0.001)	0.006*** (0.001)
Observations	436,359	436,359
Log Likelihood	−182,087.200	−182,008.900
Akaike Inf. Crit.	364,228.400	364,125.800
Bayesian Inf. Crit.	364,525.000	364,719.100
Note:		*p<0.1; **p<0.05; ***p<0.01

Table 23 shows models using different seasonal subsets of the data. Model (1) uses only the hottest 6 months of the year in each sub-region. Model (2) uses only sub-regions where the standard deviation of UTCI in the year was higher than 10. The rationale behind model (2) is to remove countries with low variation of UTCI throughout the year where the 90th percentile of UTCI may not be substantially higher than year-round averages. Standard errors are reported next to the coefficients and two-sided t-tests were conducted for a 95% confidence level. The association between a hot week and moderate-severe food insecurity holds across both models.

Table 23: Models using different seasonal subsets of the data

	Dependent variable:	
	Moderate-severe food insecurity	
	(1)	(2)
Intercept	0.101^{***} (0.022)	0.110^{***} (0.016)
Hot week	0.008^{**} (0.003)	0.005^{**} (0.002)
Area: Urban	-0.040^{***} (0.002)	-0.008^{***} (0.002)
Age	0.001^{***} (0.0001)	0.001^{***} (0.0001)
Gender: Male	-0.018^{***} (0.002)	-0.010^{***} (0.002)
Children <15 in HH	0.048^{***} (0.002)	0.026^{***} (0.002)
Having a partner	-0.027^{***} (0.002)	-0.035^{***} (0.002)
Precipitation (ln)	-0.0003^{**} (0.0002)	-0.0004^{**} (0.0002)
Year-round UTCI in subregion	0.006^{***} (0.001)	0.002^{***} (0.001)
Hot days in the last 365 days	0.0002 (0.0002)	-0.001^{***} (0.0003)
2015	0.019^{***} (0.003)	0.013^{***} (0.003)
2016	0.053^{***} (0.004)	0.018^{***} (0.003)
2017	0.082^{***} (0.004)	0.030^{***} (0.003)
Observations	172,537	128,414
Log Likelihood	−73,922.300	−24,805.990
Akaike Inf. Crit.	147,876.600	49,643.990
Bayesian Inf. Crit.	148,037.500	49,800.190
Note:		*p<0.1; **p<0.05; ***p<0.01

5.2 Short term lags

In this section, I explore the short-term lagged effects of heat on food insecurity in more detail. The effect of heat may build over several days. For example, physiological heat stress accumulates over time and households may be able to buffer a single day of productivity losses but struggle when heat persists and they miss out on several days of potential earnings.

Table 24 therefore shows two models investigating the short-term effects of heat over the last few days leading up to the survey on food insecurity. Model (1) uses the number of hot days in the 7 days leading up to and including the survey day in no particular chronological order. Model (2) uses a lagged variable for every day in the last week ordered chronologically. For example, 'Current day -1: Hot' indicates that the day before the survey date was hot, whereas '1 hot day in last week' indicates that any day in the last week was hot. Standard errors are reported next to the coefficients and two-sided t-tests were conducted for a 95% confidence level.

Model (1) shows that the effects of heat become larger and more significant as the number of hot days increases. In model (2), the current day has a positive and significant effect on moderate-severe food insecurity while all other lagged days are negative and insignificant with the exception of day -4. Both models point towards recency in the effect of heat on food insecurity by showing the accumulation effect (Model 1), and by showing that the effect of a hot day on the survey date is positive and significant when controlling for heat on the days leading up to the interview (Model 2). The models appear to indicate slightly different time frames: 1) suggests several hot days are needed, 2) at first sight seems to suggest that the current day is most relevant. However, the two models make different comparisons. Model (1) compares observations with 1,2,3,4,5,6, or 7 hot days to those who did not experience any hot days. In contrast, Model (2) compares observations from a hot day with observations without a hot day while controlling for the heat on the days leading up to the interview. This method of modelling lags can often lead to strong multi-collinearity because UTCI today is highly correlated with UTCI yesterday [2]. I therefore decide to present Model (1) in the main manuscript and suggest further research should investigate the lagged effects in more detail using time series data and methods such as distributed lag non-linear models, which unfortunately are incompatible with the panel data in this paper.

Table 24: Models using different lagged heat variables

	Dependent variable:	
	Moderate-severe food insecurity	
	(1)	(2)
Intercept	0.122^{***} (0.020)	0.121^{***} (0.020)
1 hot day in last week	-0.002 (0.002)	
2 hot day in last week	0.003 (0.002)	
3 hot day in last week	0.008^{***} (0.003)	
4 hot day in last week	-0.0002 (0.003)	
5 hot day in last week	0.012^{***} (0.004)	
6 hot day in last week	0.010^{**} (0.004)	
7 hot day in last week	0.019^{***} (0.005)	
Current day: Hot		0.010^{***} (0.002)
Current day -1: Hot		0.001 (0.002)
Current day -2: Hot		-0.001 (0.002)
Current day -3: Hot		-0.001 (0.002)
Current day -4: Hot		0.005^{**} (0.002)
Current day -5: Hot		-0.001 (0.002)
Current day -6: Hot		0.001 (0.002)
Area: Urban	-0.035^{***} (0.001)	-0.035^{***} (0.001)
Age	0.001^{***} (0.00004)	0.001^{***} (0.00004)
Gender: Male	-0.017^{***} (0.001)	-0.017^{***} (0.001)
Children <15 in HH	0.042^{***} (0.001)	0.042^{***} (0.001)
Having a partner	-0.024^{***} (0.001)	-0.024^{***} (0.001)
Precipitation (ln)	-0.0004^{***} (0.0001)	-0.0004^{***} (0.0001)
Year-round UTCI in subregion	0.006^{***} (0.001)	0.006^{***} (0.001)
Hot days in last year	0.0002 (0.0001)	0.0002 (0.0001)
2015	0.010^{***} (0.002)	0.010^{***} (0.002)
2016	0.031^{***} (0.002)	0.031^{***} (0.002)
2017	0.053^{***} (0.002)	0.053^{***} (0.002)
Observations	427,832	427,832
Log Likelihood	$-179,706.200$	$-179,706.500$
Akaike Inf. Crit.	359,456.300	359,457.000
Bayesian Inf. Crit.	359,697.600	359,698.300
Note:		*p<0.1; **p<0.05; ***p<0.01

Regression table 25 below tests time frames by comparing the effects of the last week and second-to-last week on moderate-severe food insecurity. Standard errors are reported next to the coefficients and two-sided t-tests were conducted for a 95% confidence level. Model (1) uses the number of hot days in the last week as a measure for heat. Model (2) uses the number of hot days in the second-to-last week. Model (3) uses both the number of hot days in the last week and in the second-to-last week. The effect of an additional hot day in the last week is positive and significant at 0.002 [95%-CI: 0.0011 - 0.0026, t = 4.71, p < 0.001], but the effect drops by a third to 0.001 [95%-CI: 0.0004 - 0.0012, t = 3.014, p = 0.0026] when days in the second-to-last week are considered. In model (3) using both last and second-to-last week, only days in the last week are still significant at 0.0017 [95%-CI: 0.0008 - 0.0026, t = 3.78, p < 0.001] and days in the second-to-last week at 0.0005 [95%-CI: -0.0003 - 0.0013, t = 1.067, p = 0.286]. This indicates that more recent hot days have a larger and more significant effect compared to less recent hot days.

Table 25: Models using different time frames for heat measures

	Dependent variable:		
	Moderate-severe food insecurity		
	(1)	(2)	(3)
Intercept	0.122^{***} (0.020)	0.121^{***} (0.020)	0.122^{***} (0.020)
Hot days in last week	0.002^{***} (0.0004)		0.002^{***} (0.0004)
Hot days in second to last week		0.001^{***} (0.0004)	0.0005 (0.0004)
Area: Urban	-0.035^{***} (0.001)	-0.035^{***} (0.001)	-0.035^{***} (0.001)
Age	0.001^{***} (0.00004)	0.001^{***} (0.00004)	0.001^{***} (0.00004)
Gender: Male	-0.017^{***} (0.001)	-0.017^{***} (0.001)	-0.017^{***} (0.001)
Children <15 in HH	0.042^{***} (0.001)	0.042^{***} (0.001)	0.042^{***} (0.001)
Having a partner	-0.024^{***} (0.001)	-0.024^{***} (0.001)	-0.024^{***} (0.001)
Precipitation (ln)	-0.0004^{***} (0.0001)	-0.0004^{***} (0.0001)	-0.0004^{***} (0.0001)
Year-round UTCI in subregion	0.006^{***} (0.001)	0.006^{***} (0.001)	0.006^{***} (0.001)
Hot days in last year	0.0002 (0.0001)	0.0002 (0.0001)	0.0001 (0.0001)
2015	0.010^{***} (0.002)	0.010^{***} (0.002)	0.010^{***} (0.002)
2016	0.031^{***} (0.002)	0.032^{***} (0.002)	0.031^{***} (0.002)
2017	0.053^{***} (0.002)	0.053^{***} (0.002)	0.053^{***} (0.002)
Observations	427,832	427,832	427,832
Log Likelihood	−179,686.100	−179,692.700	−179,692.400
Akaike Inf. Crit.	359,404.200	359,417.500	359,418.700
Bayesian Inf. Crit.	359,579.700	359,592.900	359,605.200
Note:			*p<0.1; **p<0.05; ***p<0.01

6 Sensitivity

6.1 Outliers

I investigate whether the results are driven by outliers. While usual sensitivity analysis analyse the influence of individual observations, this approach is not adequate here because the number of observations are large and the dependent and independent variables of interest are binary. I therefore investigate whether results are driven by observations from individual countries.

As a first step, I sequentially fit models dropping observations from one country at a time. I report the estimate and 95%-confidence intervals for the effect of a hot week on moderate-severe food insecurity in Figure 7 below. The figure shows that the estimate remains positive and significant for every dropped country.

As a second step, I conduct a DFBETA test. This test calculates the difference of the main model's estimate from the estimates of the models dropping one country at a time. These differences are then compared to the threshold calculated as $\frac{2}{\sqrt{n}} = \frac{2}{\sqrt{427{,}832}} = 0.0031$. The most influential case is Niger with a DFBETA of 0.0021. Excluding Niger from the observations reduces the coefficient the most and it drops to 0.00538 [95%-CI: 0.001878, 0.00888, t = 3.01, p = 0.0026] compared to 0.0075 [95%-CI 0.004 - 0.011, t = 4.197, p < 0.001] using a full data set. The analysis shows that even for the largest outlier, the DFBETA is well within the threshold and the model results are robust to outliers.

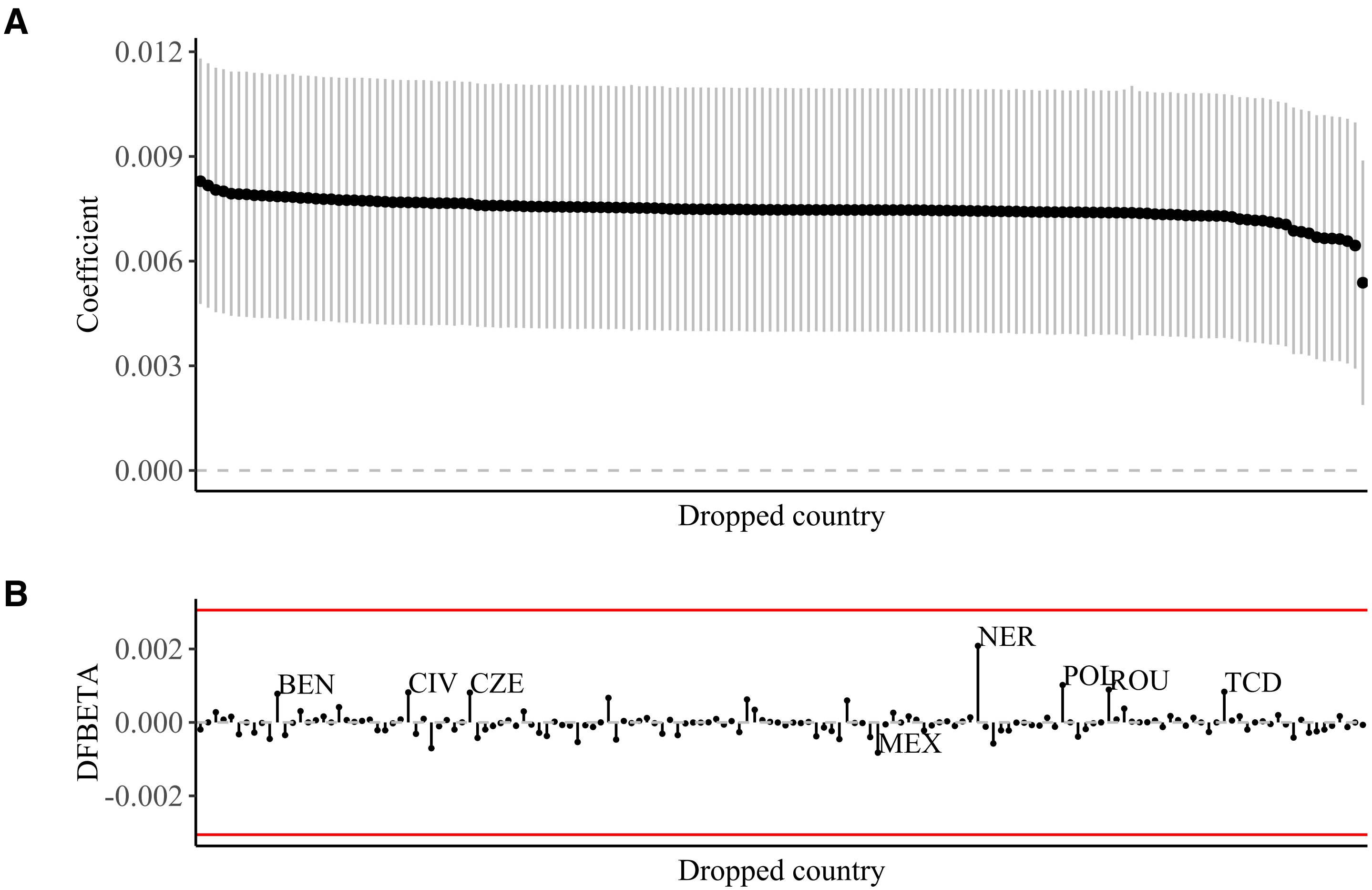

Figure 7: A) Coefficients for the association between a hot week and moderate-severe food insecurity with their 95%-confidence interval. Estimates are based on sequentially dropping individual countries from the data and retesting the association between a hot week and moderate-severe food insecurity. B) DFBETAs for the hot week coefficient from the models using data subsets and the model using the full data. The red line indicates the upper and lower threshold of 0.0031 and -0.0031. The most influential cases are labelled.

6.2 Covariates

Table 26 shows models using different sets of covariates. Model (1) uses no controls, (2) uses household-level demographic controls, and (3) uses weather-related controls. Standard errors are reported next to the coefficients and two-sided t-tests were conducted for a 95% confidence level. The association holds across all models but the coefficient grows in size when including weather-related controls.

Table 26: Models using different sets of covariates

	Dependent variable:		
	Moderate-severe food insecurity		
	(1)	(2)	(3)
Intercept	0.2914*** (0.0200)	0.2530*** (0.0197)	0.1519*** (0.0200)
Hot week	0.0054*** (0.0016)	0.0055*** (0.0016)	0.0072*** (0.0018)
Area: Urban		−0.0334*** (0.0013)	
Age		0.0011*** (0.00003)	
Partner: Yes		−0.0233*** (0.0011)	
Gender: Male		−0.0165*** (0.0010)	
Children: Yes		0.0439*** (0.0012)	
Precipitation (ln)			−0.0004*** (0.0001)
Hot days in the last 365 days			0.0002 (0.0001)
Year-round UTCI in subregion			0.0063*** (0.0005)
2015			0.0096*** (0.0017)
2016			0.0310*** (0.0017)
2017			0.0524*** (0.0017)
Number of subregions	2935	2935	2557
Number of countries	150	150	149
Standard deviation of subregions	0.0814	0.0797	0.0833
Standard deviation of countries	0.244	0.2387	0.2072
Observations	543,852	532,379	436,570
Log Likelihood	−233,823.8000	−226,559.6000	−185,265.0000
Akaike Inf. Crit.	467,657.5000	453,139.1000	370,551.9000
Bayesian Inf. Crit.	467,713.6000	453,251.0000	370,672.8000
Note:			*p<0.1; **p<0.05; ***p<0.01

References

[1] Reuben M. Baron and David A. Kenny. The moderator–mediator variable distinction in social psychological research: Conceptual, strategic, and statistical considerations. 51(6):1173–1182.

[2] Krishnan Bhaskaran, Antonio Gasparrini, Shakoor Hajat, Liam Smeeth, and Ben Armstrong. Time series regression studies in environmental epidemiology. 42(4):1187–1195.

[3] Markus C. Elze, John Gregson, Usman Baber, Elizabeth Williamson, Samantha Sartori, Roxana Mehran, Melissa Nichols, Gregg W. Stone, and Stuart J. Pocock. Comparison of propensity score methods and covariate adjustment. 69(3):345–357.

[4] Christian Fong, Chad Hazlett, and Kosuke Imai. Covariate balancing propensity score for a continuous treatment: Application to the efficacy of political advertisements. 12(1).

[5] Mahaveer Golechha, Dileep Mavalankar, and Subhash Chander Bhan. India: Heat wave and action plan implementation in indian cities. In Chao Ren and Glenn McGregor, editors, *Urban Climate Science for Planning Healthy Cities*, volume 5, pages 285–308. Springer International Publishing. Series Title: Biometeorology.

[6] Yuming Guo, Antonio Gasparrini, Ben G. Armstrong, Benjawan Tawatsupa, Aurelio Tobias, Eric Lavigne, Micheline de Sousa Zanotti Staglior Coelho, Xiaochuan Pan, Ho Kim, Masahiro Hashizume, Yasushi Honda, Yue-Liang Leon Guo, Chang-Fu Wu, Antonella Zanobetti, Joel D. Schwartz, Michelle L. Bell, Matteo Scortichini, Paola Michelozzi, Kornwipa Punnasiri, Shanshan Li, Linwei Tian, Samuel David Osorio Garcia, Xerxes Seposo, Ala Overcenco, Ariana Zeka, Patrick Goodman, Tran Ngoc Dang, Do Van Dung, Fatemeh Mayvaneh, Paulo Hilario Nascimento Saldiva, Gail Williams, and Shilu Tong. Heat wave and mortality: A multicountry, multicommunity study. 125(8):087006.

[7] Kosuke Imai, Luke Keele, and Dustin Tingley. A general approach to causal mediation analysis. 15(4):309–334.

[8] Kosuke Imai, Luke Keele, and Teppei Yamamoto. Identification, inference and sensitivity analysis for causal mediation effects. 25(1).

[9] Pierre Masselot, Malcolm Mistry, Jacopo Vanoli, Rochelle Schneider, Tamara Iungman, David Garcia-Leon, Juan-Carlos Ciscar, Luc Feyen, Hans Orru, Aleš Urban, Susanne Breitner, Veronika Huber, Alexandra Schneider, Evangelia Samoli, Massimo Stafoggia, Francesca de'Donato, Shilpa Rao, Ben Armstrong, Mark Nieuwenhuijsen, Ana Maria Vicedo-Cabrera, Antonio Gasparrini, Souzana Achilleos, Jan Kyselý, Ene Indermitte, Jouni J.K. Jaakkola, Niilo Ryti, Mathilde Pascal, Klea Katsouyanni, Antonis Analitis, Patrick Goodman, Ariana Zeka, Paola Michelozzi, Danny Houthuijs, Caroline Ameling, Shilpa Rao, Susana das Neves Pereira da Silva, Joana Madureira, Iulian-Horia Holobaca, Aurelio Tobias, Carmen Íñiguez, Bertil Forsberg, Christofer Åström, Martina S. Ragettli, Antonis Analitis, Klea Katsouyanni, First name Surname, Sofia Zafeiratou, Liliana Vazquez Fernandez, Ana Monteiro, Masna Rai, Siqi Zhang, and Kristin Aunan. Excess mortality attributed to heat and cold: a health impact assessment study in 854 cities in europe. 7(4):e271–e281.

[10] Antonella Mazzone and Radhika Khosla. Socially constructed or physiologically informed? placing humans at the core of understanding cooling needs. 77:102088.

[11] Cassandra W. Pattanayak, Donald B. Rubin, and Elizabeth R. Zell. Métodos de puntuación de propensión para crear una distribución equilibrada de las covariables en los estudios observacionales. 64(10):897–903.

[12] Marina Romanello, Alice McGushin, Claudia Di Napoli, Paul Drummond, Nick Hughes, Louis Jamart, Harry Kennard, Pete Lampard, Baltazar Solano Rodriguez, Nigel Arnell, Sonja Ayeb-Karlsson, Kristine Belesova, Wenjia Cai, Diarmid Campbell-Lendrum, Stuart Capstick, Jonathan Chambers, Lingzhi Chu, Luisa Ciampi, Carole Dalin, Niheer Dasandi, Shouro Dasgupta, Michael Davies, Paula Dominguez-Salas, Robert Dubrow, Kristie L Ebi, Matthew Eckelman, Paul Ekins, Luis E Escobar, Lucien Georgeson, Delia Grace, Hilary Graham, Samuel H Gunther, Stella Hartinger, Kehan He, Clare Heaviside, Jeremy Hess, Shih-Che Hsu, Slava Jankin, Marcia P Jimenez, Ilan Kelman, Gregor Kiesewetter, Patrick L Kinney, Tord Kjellstrom, Dominic Kniveton, Jason K W Lee, Bruno Lemke, Yang Liu, Zhao Liu, Melissa Lott, Rachel Lowe, Jaime Martinez-Urtaza, Mark Maslin, Lucy McAllister, Celia McMichael, Zhifu Mi, James Milner, Kelton Minor, Nahid Mohajeri, Maziar Moradi-Lakeh, Karyn Morrissey, Simon Munzert, Kris A Murray, Tara Neville, Maria Nilsson, Nick Obradovich,

Maquins Odhiambo Sewe, Tadj Oreszczyn, Matthias Otto, Fereidoon Owfi, Olivia Pearman, David Pencheon, Mahnaz Rabbaniha, Elizabeth Robinson, Joacim Rocklöv, Renee N Salas, Jan C Semenza, Jodi Sherman, Liuhua Shi, Marco Springmann, Meisam Tabatabaei, Jonathon Taylor, Joaquin Trinanes, Joy Shumake-Guillemot, Bryan Vu, Fabian Wagner, Paul Wilkinson, Matthew Winning, Marisol Yglesias, Shihui Zhang, Peng Gong, Hugh Montgomery, Anthony Costello, and Ian Hamilton. The 2021 report of the lancet countdown on health and climate change: code red for a healthy future. 398(10311):1619–1662.

[13] Ambarish Vaidyanathan, Shubhayu Saha, Ana M. Vicedo-Cabrera, Antonio Gasparrini, Nabill Abdurehman, Richard Jordan, Michelle Hawkins, Jeremy Hess, and Anne Elixhauser. Assessment of extreme heat and hospitalizations to inform early warning systems. 116(12):5420–5427.
